# Stereoselective synthesis of a 4-⍺-glucoside of valienamine and its X-ray structure in complex with *Streptomyces coelicolor* GlgE1-V279S

**DOI:** 10.1038/s41598-021-92554-9

**Published:** 2021-06-28

**Authors:** Anshupriya Si, Thilina D. Jayasinghe, Radhika Thanvi, Sunayana Kapil, Donald R. Ronning, Steven J. Sucheck

**Affiliations:** 1 Department of Chemistry and Biochemistry, University of Toledo, Toledo, OH 43606 USA; 2 Department of Pharmaceutical Sciences, University of Nebraska Medical Center, Omaha, NE 68198 USA; 3 Merck through ExecuPharm, Rahway, NJ 07065 USA

**Keywords:** Structural biology, X-ray crystallography, Biochemistry, Drug discovery, Medicinal chemistry, Structure-based drug design, Chemistry, Organic chemistry, Carbohydrate chemistry

## Abstract

Glycoside hydrolases (GH) are a large family of hydrolytic enzymes found in all domains of life. As such, they control a plethora of normal and pathogenic biological functions. Thus, understanding selective inhibition of GH enzymes at the atomic level can lead to the identification of new classes of therapeutics. In these studies, we identified a 4-⍺-glucoside of valienamine (**8**) as an inhibitor of *Streptomyces coelicolor* (*Sco*) GlgE1-V279S which belongs to the GH13 Carbohydrate Active EnZyme family. The results obtained from the dose–response experiments show that **8** at a concentration of 1000 µM reduced the enzyme activity of *Sco* GlgE1-V279S by 65%. The synthetic route to **8** and a closely related 4-⍺-glucoside of validamine (**7**) was achieved starting from readily available D-maltose. A key step in the synthesis was a chelation-controlled addition of vinylmagnesium bromide to a maltose-derived enone intermediate. X-ray structures of both **7** and **8** in complex with *Sco* GlgE1-V279S were solved to resolutions of 1.75 and 1.83 Å, respectively. Structural analysis revealed the valienamine derivative **8** binds the enzyme in an E_2_ conformation for the cyclohexene fragment. Also, the cyclohexene fragment shows a new hydrogen-bonding contact from the pseudo-diaxial C(3)–OH to the catalytic nucleophile Asp 394 at the enzyme active site. Asp 394, in fact, forms a bidentate interaction with both the C(3)–OH and C(7)-OH of the inhibitor. In contrast, compound **7** disrupts the catalytic sidechain interaction network of *Sco* GlgE1-V279S via steric interactions resulting in a conformation change in Asp 394. These findings will have implications for the design other aminocarbasugar-based GH13-inhibitors and will be useful for identifying more potent and selective inhibitors.

## Introduction

**Figure 1:**
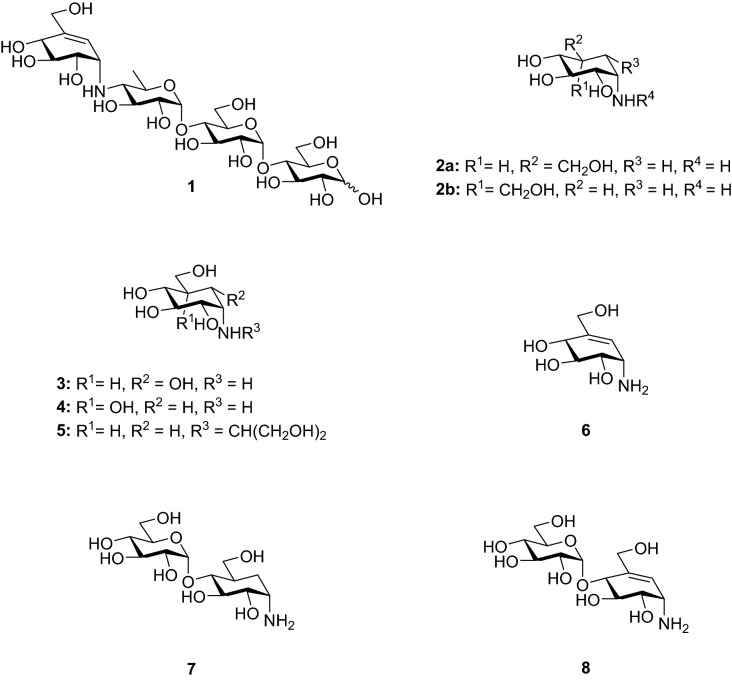
Aminocarbasugar-based GH inhibitors and targets **7** and **8**.

Glycoside hydrolase (GH) enzymes are hydrolases involved in a large array of biological phenomena^1–3^. Inhibiting the GH activity to alter the glycosylation or catabolism of glycans is an important area of inquiry for the development of new glycomimetics^4–7^. An important class of GH inhibitors in glycobiology are the carbasugars, wherein the endocyclic ring oxygen is replaced by a methylene group or an *sp*^2^ hybridized carbon^8,9^. Some of these pseudosaccharides may mimic the transition state (TS) of the catalyzed reaction while others may mimic the Michaelis complex^4^. The exact nature of GH inhibition by carbasugars remains complex as to whether they are actual TS inhibitors or merely tight binders^10–13^. 1-Aminocarbasugars are thought to mimic charge development in the TS by incorporating a basic nitrogen atom in place of C(1)–O^14^. One example of this compound class is Acarbose (**1**), an inhibitor of the GH13 enzyme human pancreatic α-amylase and a treatment for type-II diabetes mellitus (T2D), Fig. 1^15,16^. The inhibitory activities of **1** and related compounds has been commonly ascribed to the ability to mimic a glucopyranosyl half-chair conformation intermediate of glycosidase catalysis^12,15,17^; further, the exocyclic nitrogen atom has been shown to form coulombic interactions in GH complexes^18^. Closely related carbasugars have been postulated to form an initial Michaelis complex with GHs in the ^2^H_3_ conformation^19^.

In addition to ionic interactions, it is important to appreciate the conformational changes substrates undergo during enzymatic catalysis and there is limited information on the conformational itinerary of GH13 substrates^20,21^. Data on different GH13 enzymes suggest a conformational itineraries consistent with ^4^C_1_ → ^4^H_3_-TS → ^1^S_3_^21^ or possibly ^4^C_1_ → ^1^S_3_ → ^4^H_3_-TS → ^4^C_1_^22^. In addition, computational studies indicate the possibility of ^4^C_1_ → ^4^H_3_-TS → ^4^C_1_ for the first half-reaction and E_3_ → E_3_-TS → ^4^C_1_ for the second half-reaction for amylosucrase^23^. Thus, the ^4^H_3_-TS is believed constant among GH13 enzymes. Earlier, kinetic isotope effect studies have suggested a ^2,5^B TS could also be at play for a yeast α-glucosidase^24^. Taking these conformational itineraries into consideration in the inhibitor design is expected to enhance inhibitor-enzyme interactions. The importance of this line of inquiry can be seen by the vigorous exploration of both covalent^25–27^ and non-convent inhibitors for this class of enzymes^28^.

Aminocarbasugars related to validamine (**2a**) and valienamine (**6**)^29,30^ show inhibitory activity against α-GHs such as α-amylase, rice α-glucosidase and yeast α-glucosidase^31,32^. IC_50_ values for compounds **2**–**6** against α-amylase are in the range of 10^−2^ mol/L and against yeast α-glucosidase is between 10^−5^–10^−4^ mol/L^31^. Voglibose (**5**), a derivative of **2a**, has also found use as an α-glucosidase inhibitor and treatment for T2D^32^. Based on these reports, we proposed the 4-⍺-glucoside of validamine (**7**) and 4-⍺-glucoside of valienamine (**8**) would be potential inhibitors of α-GHs that can accommodate an α-configured − 2 site sugar, for example, *Sco* GlgE1-V279S, Fig. 1.

**Figure 2:**
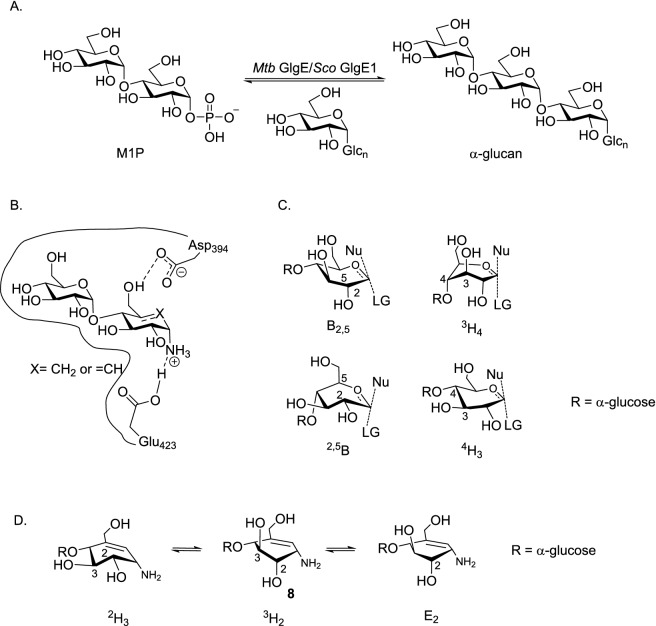
(**A**) *Mtb* GlgE/*Sco* GlgE1 catalyzed glycosylation of α-1,4 glucan with M1P. (**B**) Proposed *Sco* GlgE1-V279S/**7** and *Sco* GlgE1-V279S/**8** complex coulombic interactions. (**C**) B_2,5_, ^2,5^B, ^4^H_3_, and ^3^H_4_ C5–O5–C1–C2 planar TS conformations commonly invoked for GH. (**D**) Possible conformations of the cyclohexene fragment of **8**.

The GlgE homologs of Streptomyces and Mycobacteria catalyze the conversion of maltose-1-phosphate (M1P) to linear α-(1 → 4) glucans which play an important role in the bacterial cell wall formation, Fig. 2A ^33^. *Mtb* GlgE is a genetically validated anti-TB target as the absence of *Mtb* GlgE leads to cytosolic accumulation of toxic levels of M1P by a self-amplifying feedback response leading to cell death^33^. Previously, we have shown that hydroxypyrrolidines modified with an α-configured − 2 site sugar can inhibit *Mtb* GlgE (*K*_i_ = 237 ± 27 µM) as well *Sco* GlgE1-V279S (*K*_i_ = 102 ± 7 µM), a closely related homolog from *Streptomyces coelicolor* (*Sco*) GlgE1 that has been mutated to be 100% identical to the M1P binding site of *Mtb* GlgE, and affords much higher quality X-ray diffraction data^34^.

Keeping in mind that *Sco* GlgE1 is an α-retaining glycoside hydrolase-like phosphorylase that uses a two-step substitution mechanism involving participation by an enzymatic nucleophile and general acid^35,36^, we proposed 1-aminocarbasugar **7** or **8** could form either Michaelis-like or TS-like contacts with *Sco* GlgE1-V279S and would form favorable coulombic interactions with the Glu 432 general acid, Fig. 2B. In addition, it is appreciated that the TSs for GH are considered to have oxocarbenium-like character. It is hypothesized that the developing charge is stabilized via partial double bond character between C1 and the endocyclic oxygen. As a result, maximal stabilization is expected when C5–O5–C1–C2 are planar^37^. Based on that argument, it has been noted that: B_2,5_, ^2,5^B, ^4^H_3_, and ^3^H_4_ pyranose conformations as well as E_3_ and ^3^E afford these structural requirements^38^, and frequently represent TSs for GH, Fig. 2C. The ^4^H_3_ conformation is the generally accepted transition state for GH13 enzymes^22^. While compound **7** or **8** cannot completely mimic this TSs, compound **8** can exist in a ^2^H_3_ conformation or ^3^H_2_ conformation, Fig. 2D. Also, higher energy conformations such as E_2_ may be accessed depending on binding forces that could, and do (*vide infra*), develop in an enzyme-inhibitor complex. Of these, the ^2^H_3_ conformation of **8** was considered as close to a ^4^H_3_ conformation. In addition, unsaturated cyclitol ethers are known to be accepted as substrates by α-amylases^39^. Consequently, our target carbasugars **7** and **8** were synthesized, evaluated for inhibition against *Sco* GlgE1-V279S, and their X-ray crystal structures solved in complex with *Sco* GlgE1-V279S to characterize the lowest energy conformations stabilized through interactions within the enzyme active site.

Previously, Tadashi et al. reported the enzymatic syntheses of α- and β-glucoside derivatives of validamine (**2a**) and valienamine (**6**) using maltose as a glucose donor in a glucosidase catalyzed transglycosylation^29,30^. The acceptors **2a** and **6** were obtained via microbial degradation of validamycin A by culturing soil bacteria *Pseudomonas denitrificans*^40^ and *Flavobacterium saccharophilum*^41^, respectively, and isolating the products by ion-exchange chromatography. α- and β-glucosidases were extracted from *Rhodotorula lactosa*, purified by carboxymethyl (CM)-cellulose chromatography and used for catalyzing the α- and β-transglucosidations, respectively. 7-α-Isomaltoside and 4-α-glucoside (**7**) from **2a** and 7-α-glucoside, 7-α-isomaltoside, 4-α-isomaltoside and 4-α-glucoside (**8**) from **6** were some of the products obtained from these transglucosidation reactions. The inhibitory test results of these derivatives against various α-glucosidases like rat intestinal maltase, sucrase, glucoamylase, isomaltase and trehalase suggested that **2a**, **6**, **7** and **8** (IC_50_ = 10^−5^ to 10^−2^ M) represent reasonable starting points for the development of more potent inhibitors^29,30^.

**Scheme 1:**
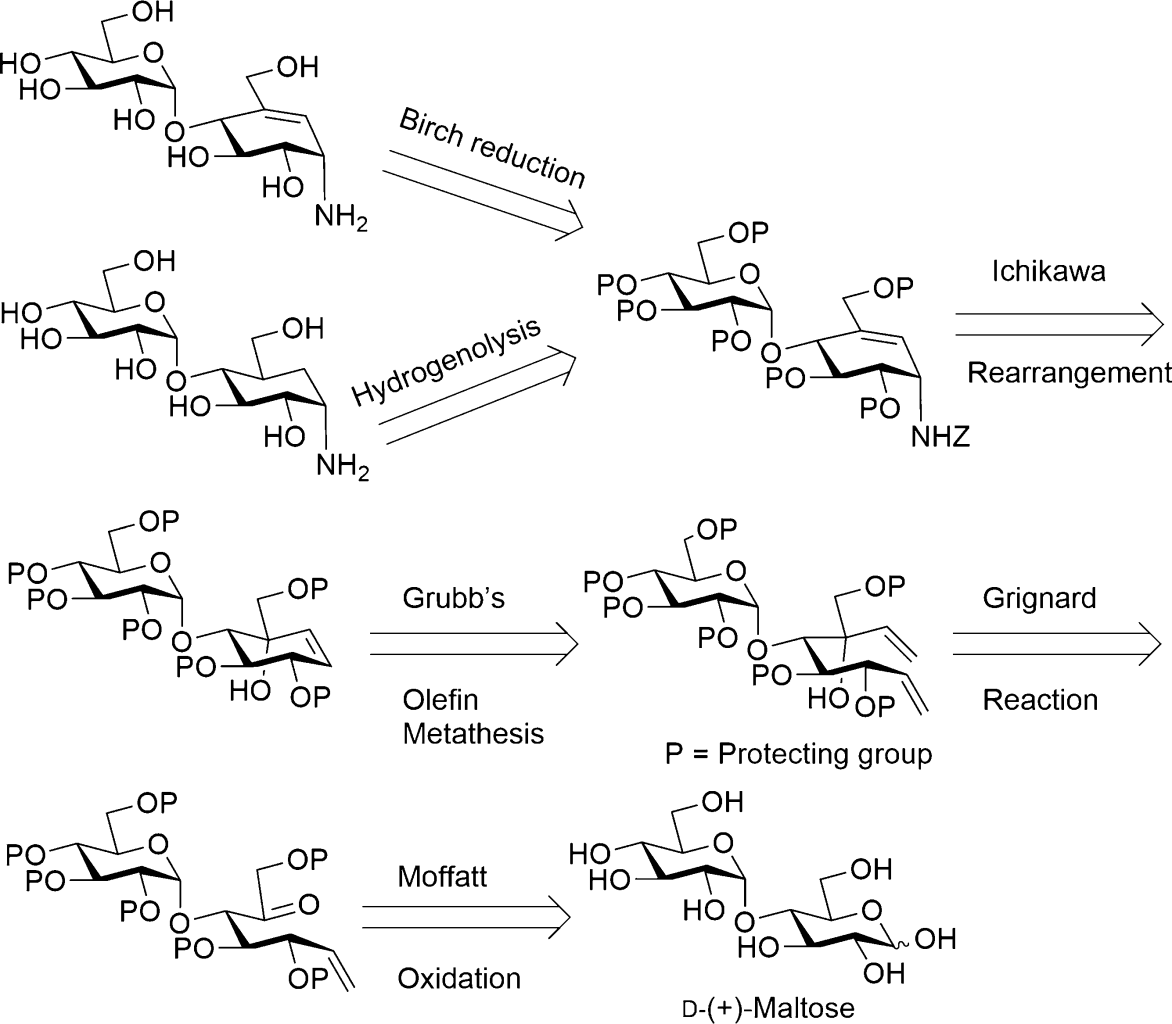
Retrosynthetic strategy to prepare 4-α-glycoside derivatives of valienamine and validamine from maltose.

However, relying on natural products and enzymatic methods to obtain **7** and **8** is very challenging due to the availability of biological sources, complex purifications, and complex spectroscopic characterization of closely related regioisomers. In order to overcome these difficulties, the development of an efficient synthetic strategy for the aminocarbasugars **7** and **8** was developed. In 1999, Kapferer et al. reported a practical synthetic route to the formation of valienamine (**6**) that involved a stereoselective addition of vinyl magnesium bromide to give separable dienes followed by a ring closing metathesis to give a cyclohexene adduct which could be transformed to the desired **6**^42^. In this work, we applied this method to the regiospecific synthesis of aminocarbasugars **7** and **8**. Scheme 1 illustrates the retrosynthetic route for the desired target inhibitors. Compounds **7** and **8** were then prepared in twelve steps using commercially available D-(+)-maltose as the starting material.

## Results and discussion

**Scheme 2:**
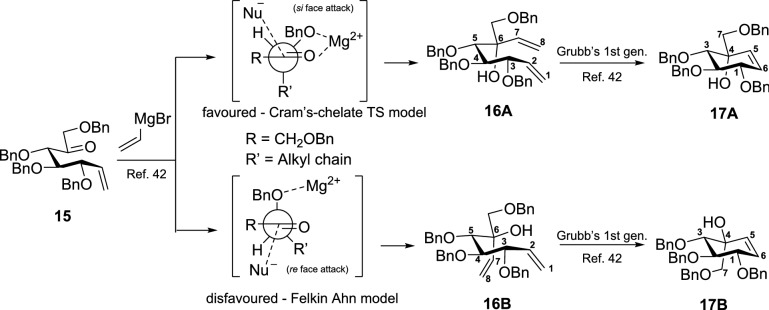
Diastereoselective addition of vinylmagnesium bromide to enone **15**.

**Figure 3:**
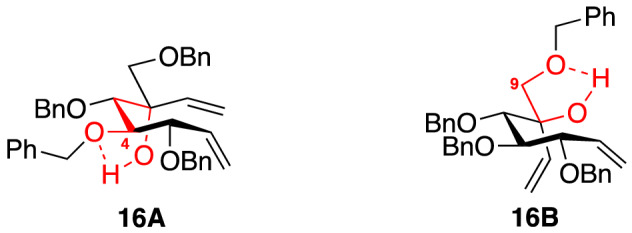
Intramolecular H-bonding interactions of the diastereomers **16A**, **27A**, **16B** and **27B**.

Before synthesizing the target pseudodisaccharides, we synthesized valienamine (**6**) from commercially available D-(+)-glucose (**9**) to obtain spectroscopic diastereoselectivity metrics for the intermediates afforded by the stereoselective addition of vinylmagnesium bromide to ketone **15**, Scheme 2. The spectral analysis of the intermediates **16A**, **16B**, **17A**, and **17B** proved critical in analyzing the desired diastereomer of the maltosyl analogues. Compound **15** was obtained starting from **9** in seven steps that involved Wittig olefination and Moffatt oxidation (Scheme S1 in Supporting Information)^34,43,44^. The key step in the synthesis is the diastereoselective addition of vinylmagnesium bromide to enone **15** to afford separable diastereomers **16A** and **16B** (9:1). The high selectivity for the conformer **16A** over the conformer **16B** can be explained by Cram’s-chelate TS model^45,46^. According to previous reports, the torsional strain in enone **15** implies a staggered conformation in the TS where the nucleophile attacks preferentially from the *si* face, Scheme 2^42,45,47^. Moreover, the downfield shift of the C(6)–OH signal of **16A** relative to the chemical shift of C(6)–OH of **16B** indicated a strong intramolecular H-bond (six-membered ring) to C(4)–OBn in **16A** and a five-membered H-bond to C(9)–OBn in **16B**, Fig. 3.

Although, there is little to distinguish the relative stereochemistry of **16A** and **16B**, after the intramolecular ring closing metathesis (RCM), Kapferer et al^42^ assigned the absolute stereochemistry of the carbasaccharides **17A** and **17B** by comparing the spectroscopic data and Nuclear Overhauser Effect (NOE) experiments. Finally, **17A** was converted to valienamine (**6**) in three steps involving Ichikawa rearrangement, (Scheme S1 in Supporting Information)^48^. The spectral data of all the intermediates were found nearly identical to the previously reported data (Supporting Information)^42,49^.

**Scheme 3:**
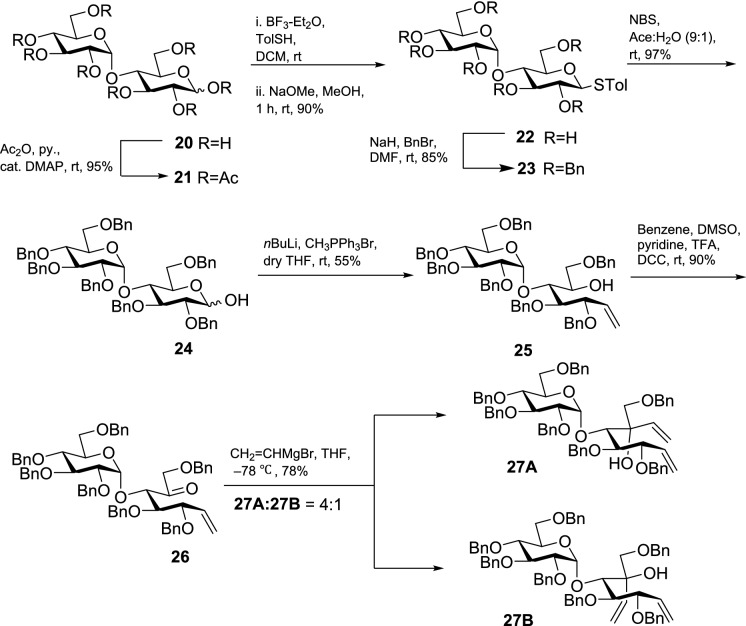
Synthesis of diastereomers **27A** and **27B** from D-(+)-maltose (**20**).

After successfully synthesizing valienamine (**6**), we synthesized the pseudodisaccharides **7** and **8** starting from D-(+)-maltose, Scheme 3, and the intermediates of Scheme 2 were used to assign the diastereomers. D-(+)-maltose (**20**) was peracetylated with acetic anhydride followed by the treatment with *p*-thiocresol and boron-trifluoride diethyl etherate to generate a peracetylated β-thiomaltoside^50^. Subsequently, deacetylation under Zemplén conditions afforded glycoside **22** followed by benzylation to afford perbenzylated thiomaltoside (**23**) in 85% yield^51^. We treated thiomaltoside **23** with *N*-bromosuccinimide (NBS) in acetone:water (9:1) to obtain the reducing sugar **24**^52^. Wittig methylenation of **24** using methyltriphenylphosphonium bromide in the presence of *n*-butyl lithium afforded alkene **25** in 55% yield^53^. After that, the secondary alcohol **25** was oxidized under Moffatt oxidation conditions to afford ketone **26** in 90% yield^44^. Compound **26** was subjected to the stereoselective addition using vinylmagnesium bromide in dry THF to afford diastereomeric dienes **27A** and **27B** in a 4:1 ratio, respectively (Scheme 3).

The ^1^H NMR chemical shift values and coupling constants of the epimers **27A** and **27B** were found to be comparable with the ^1^H NMR chemical shift values and the coupling constants of the epimers **16A** and **16B** (Table 1). Although the chemical shift of the terminal alkenyl protons in **27A** (Ha–C(8) 5.59 ppm, Hb–C(8) 5.27 ppm) and **27B** (Ha–C(8) 5.60 ppm, Hb–C(8) 5.30 ppm) appear in the same range as the respective terminal alkenyl protons in **16A** (Ha–C(8) 5.47 ppm, Hb–C(8) 5.19 ppm), and **16B** (Ha–C(8) 5.45 ppm, Hb–C(8) 5.23 ppm) the C(6)–OH signals of the maltosyl diastereomers followed the same trend as the C(6)–OH signals of the glucosyl diastereomers *vide supra*. The observed *J*(9a, 9b = 8.9 Hz) values for **27A** are smaller than the *J*(9a, 9b = 9.2 Hz) values for **27B**. Indeed, a similar pattern is noticed in the glucosyl dienes suggesting that **27A** and **27B** possess the same conformation as **16A** *J*(9a, 9b = 8.7 Hz) and **16B** *J*(9a, 9b = 9.3 Hz) respectively. However, the exact stereochemistry of the diastereomers **27A** and **27B** could not be confidently confirmed using only the ^1^H NMR chemical shift and *J* values.

**Table 1:**
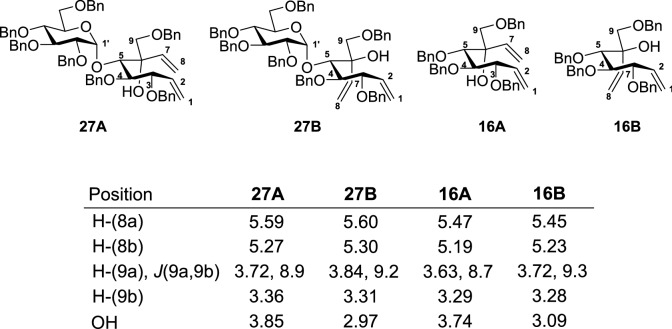
Selected ^1^H NMR shift values (ppm) of compound **27A**, **27B**, **16A**, and **16B**.

**Scheme 4:**
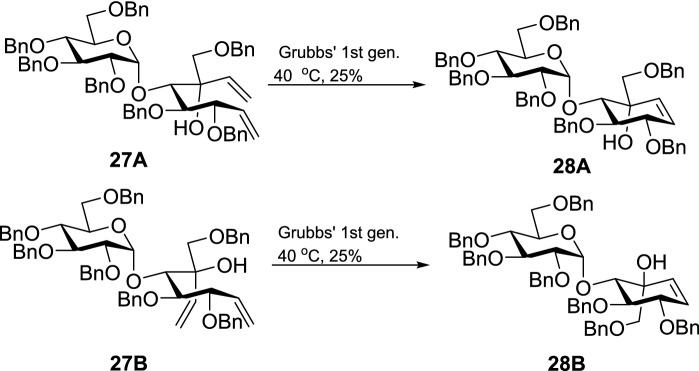
RCM of dienes **27A** and **27B**.

Complete structure elucidation of the desired epimer was achieved by further subjecting the dienes **27A** and **27B** to RCM using Grubbs’ first-generation catalyst, Scheme 4. The reaction was optimized by using various conditions to improve the yield, Table 2. The highest yield (25%) was obtained using Grubbs’ first-generation catalyst under DCM reflux conditions.

**Table 2:** Reaction condition optimization for RCM for compounds **28A** and **28B**.

Catalyst (10 mol%)	Temperature	Solvent	Yield
Grubbs’ 2nd gen	rt	DCM	NR
Grubbs’ 2nd gen	40 °C	DCM	NR
Hoveyda Grubbs’	rt	DCM	NR
Grubbs’ 1st gen	rt	DCM	10–15%
Grubbs’ 1st gen	40 °C	DCM	25%
Grubbs’ 1st gen	rt	Toluene	14%
Grubbs’ 1st gen	80 °C	Toluene	15%

The ^1^H NMR chemical shift values of **28A** and **28B** were compared with the α-glycosylated aminocarbasugars **17A** and **17B**. The alkenyl protons in **28A** (H–C(5) 5.69 ppm, H–C(6) 5.92 ppm) and **28B** (H–C(5) 5.74 ppm, H–C(6) 5.74 ppm) appear at the exact same chemical shifts as the respective alkenyl protons in **17A** (H–C(5) 5.69 ppm, H–C(6) 5.96 ppm), and **17B** (H–C(5) 5.74 ppm, H–C(6) 5.74 ppm) giving a clear indication of matching relative configurations as per literature^42^. The C-atoms carrying an equatorial substituent, especially oxy groups, resonate at a higher field than those with axial substituents, justifying the ^13^C NMR chemical shifts for C(4) of **28A** (73.10 ppm) and **28B** (75.07 ppm) (Table 3)^54^. A similar trend in the ^13^C NMR chemical shift values was also observed in the glucosyl diastereomers.

**Table 3:**
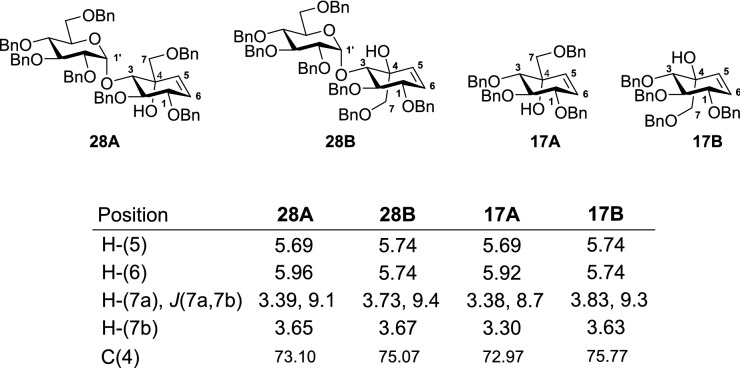
Comparing the NMR shift values of compound **28A**, **28B**, **17A** and **17B**.

**Scheme 5:**
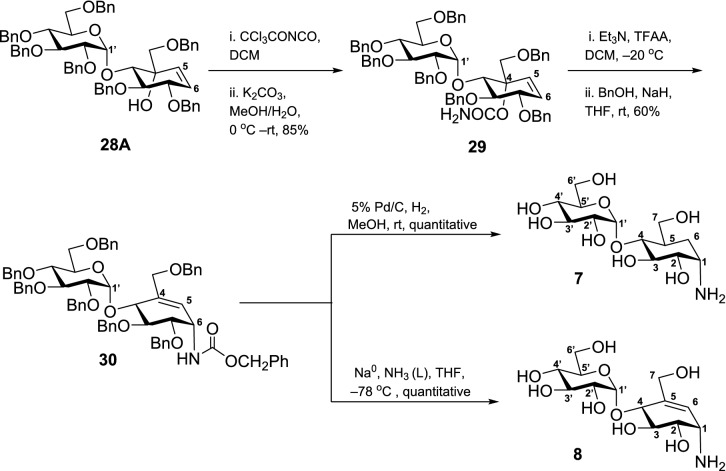
Synthesis of 4-α-glycoside derivatives of validamine **7** and valienamine **8** from alkene **28A**.

The synthesis continued with the protection of the tertiary alcohol of **28A** with trichloroacetyl isocyanate in dichloromethane (DCM) at 0 °C, followed by hydrolysis with K_2_CO_3_ in aqueous MeOH giving an 85% yield of the carbamate **29**. Compound **29** was subjected to an Ichikawa rearrangement^48^ that proved useful in the synthesis of valienamine (**6**)^42^. Here, the dehydration of **29** with trifluoroacetic anhydride (TFAA) and Et_3_N at − 20 °C formed an intermediate allylic cyanate that underwent a spontaneous [3]-sigmatropic rearrangement to afford the isocyanate. The isocyanate was treated in situ with 60% NaH in mineral oil and benzyl alcohol solution to yield protected maltosyl valienamine analog **30** in 60% overall yield, Scheme 5.

Kozikowski et al^55^ reported that hydrogenation of substituted cyclohexenes yields two diastereomers where the major isomer has the hydroxy methylene at the equatorial position. However, hydrogenation of **30** using 5 wt. % Pd/C afforded a single isomer **7** in a quantitative yield. We compared the ^1^H NMR chemical shift values of the obtained compound with reported validamine (**2a**)^56^ and *epi*-validamine (**2b**)^57^ to determine the stereochemistry at C(5), Table 4. Although the proton chemical shift value of **7** at C(5) is similar to **2b**, no sound conclusion can be derived due to difference in the chemical environment. Therefore, we compared the C(6) protons of **7** with the C(6) protons of **2a** and **2b**. For *epi*-validamine, the Ha-(6) and Hb-(6) appear at different chemical shifts (1.78 ppm and 2.11 ppm) whereas for validamine (**2a**), both the C(6) protons appear as a multiplet (1.74–1.59 ppm). After the spectral analysis of **7**, we observed a similarity in the chemical shifts and peak splitting pattern of the C(6) protons (1.71–1.55 ppm) to validamine (**2a**), thereby, confirming that the hydroxy methylene in target compound **7** is at the equatorial position. Finally, the benzyl carbamate **30** was subjected to deprotection under Birch reduction conditions to afford target compound **8**.

**Table 4:**
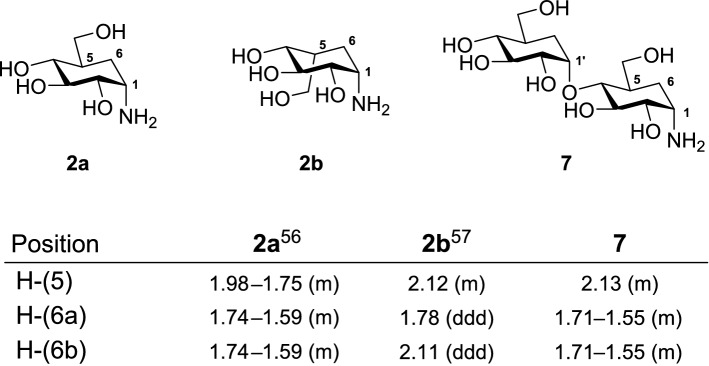
Comparing the NMR shift values of validamine (**2a**), *epi*-validamine (**2b**) and **7**.

Inhibition studies were carried out using **7** and **8** separately against *Sco* GlgE1-V279S. The maltosyltransferase activity of *Sco* GlgE1-V279S using M1P was determined by coupling with purine nucleoside phosphorylase (PNP), using the previously published method^34^. As for previous inhibitory studies, assays employed M1P at a concentration of 250 µM. Enzyme activity of *Sco* GlgE1-V279S decreased by 32% at 100 µM concentration of **8**, but **7** failed to exhibit any inhibition of *Sco* GlgE1-V279S. Similarly, a 1 mM concentration of **7** also failed to inhibit the enzyme. Therefore, only compound **8** was used for dose-dependence studies at concentrations between 0 and 1000 µM. As shown in Table 5, the presence of 750 µM and 1000 µM concentrations of **8** reduced the enzyme activity by 55% and 65%, respectively. Unfortunately, the inability to test **8** beyond 1 mM prevents inhibitor saturation of *Sco* GlgE1-V279S and a more accurate IC_50_ determination of the compound.

**Table 5:** Concentration of **8** vs **%** activity of the enzyme.

8 (µM)	% (enzyme activity ± SD)
0	100.0 ± 4.7
50	79.9 ± 1.4
100	68.5 ± 3.7
250	58.4 ± 3.3
500	54.9 ± 4.6
750	45.4 ± 1.8
1000	35.2 ± 3.5

The X-ray crystal structures of *Sco* GlgE1-V279S/**7** and Sco GlgE1-V279S/**8** complexes (accession codes: 7MEL and 7MGY, respectively and Table S1 in Supporting Information) were pursued to gain insight regarding the lack of efficacy of **7** and evaluate the role of ring flattening in affording the inhibitory activity of **8**. Although **7** failed to inhibit the activity of *Sco* GlgE1-V279S in the reaction conditions described here, we thought to examine any interactions formed between **7** and the enzyme that may inform future drug design by identifying interactions between enzyme and ligand that destabilize the *Sco* GlgE1-V279S/**7** complex. Therefore, a *Sco* GlgE1-V279S/**7** complex was prepared using 10 mM concentration of **7** and 8 mg/mL concentration of *Sco* GlgE1-V279S in the final protein-compound mixture. The formed crystals were soaked with an additional amount of **7** for additional 12 h prior to flash cooling. The final concentration of compound **7** in the drop was approximately 15 mM. The exact concentration is unknown due to changes in drop volume because of vapor diffusion between the crystallization drop and the reservoir.

**Figure 4:**
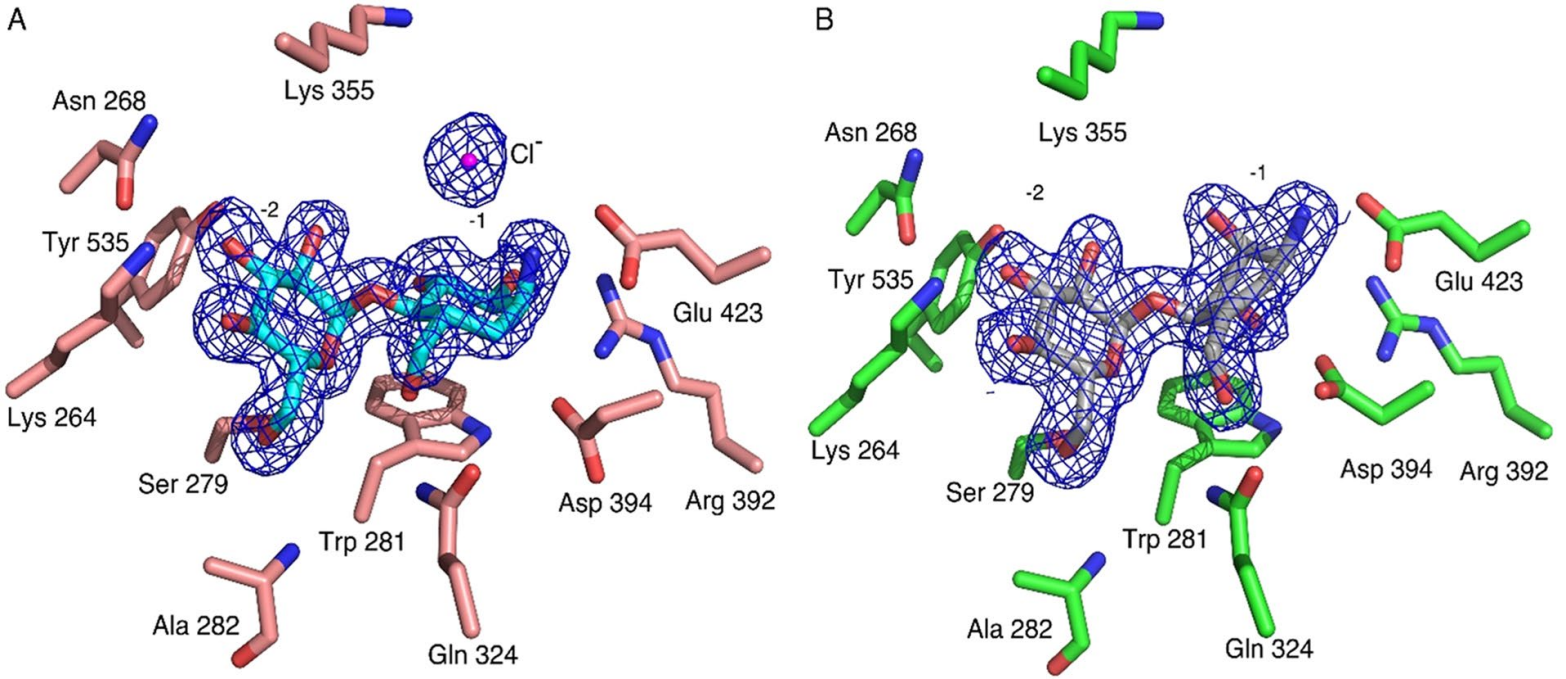
Compounds **7** and **8** bound in the active site of *Sco* GlgE1-V279S. (**A**) Complex of *Sco* GlgE1-V279S with **7**. The magenta sphere represents the ordered chloride ion. (**B**) Complex of *Sco* GlgE1-V279S with **8**. Both 2F_O_ − F_C_ composite omit maps are contoured at 1.5σ.

The results of the X-ray diffraction experiments afforded refinement of the *Sco* GlgE1-V279S/**7** and *Sco* GlgE1-V279S/**8** complex structures to resolutions of 1.75 Å and 1.83 Å, respectively. The evidence from the 2F_O_-F_C_ composite omit maps (Fig. 4) demonstrate the presence of each compound in the M1P binding site. In addition to the strong density observed for both **7** and **8** in the respective structures, the GlgE1-V279S/**7** complex also exhibits a well-ordered spherical density located 3.2 Å from the exocyclic amine moiety of **7** that we have modeled as a chloride ion. The location of the chloride ion overlaps significantly with that of negatively charged phosphate and phosphonate moieties in previous GlgE1-V279S crystal structures. Based on the proximity of the C(1) ammonium moiety to Glu 423 in both structures, the presence of the chloride ion in the *Sco* GlgE1-V279S/**7** complex structure, and the pH of the crystallization condition (pH 7.5), we expect the “amine” moiety of **7** (and **8**) to harbor a positively charged ammonium moiety and the following discussion will reflect that assumption.

The compounds bound within the M1P binding sites in each *Sco* GlgE1-V279S structure and provide important information about enzyme-compound interactions and the geometry of the compounds in that bound state. In both enzyme-compound structures, the interactions of the glucose moiety bound within the − 2 subsite are the same as that observed in previously published *Sco* GlgE1-V279S/inhibitor complex structures^58–61^. However, interactions between the carbocycle moieties and the amino acid residues forming the − 1 subsite differ significantly from each other.

**Figure 5:**
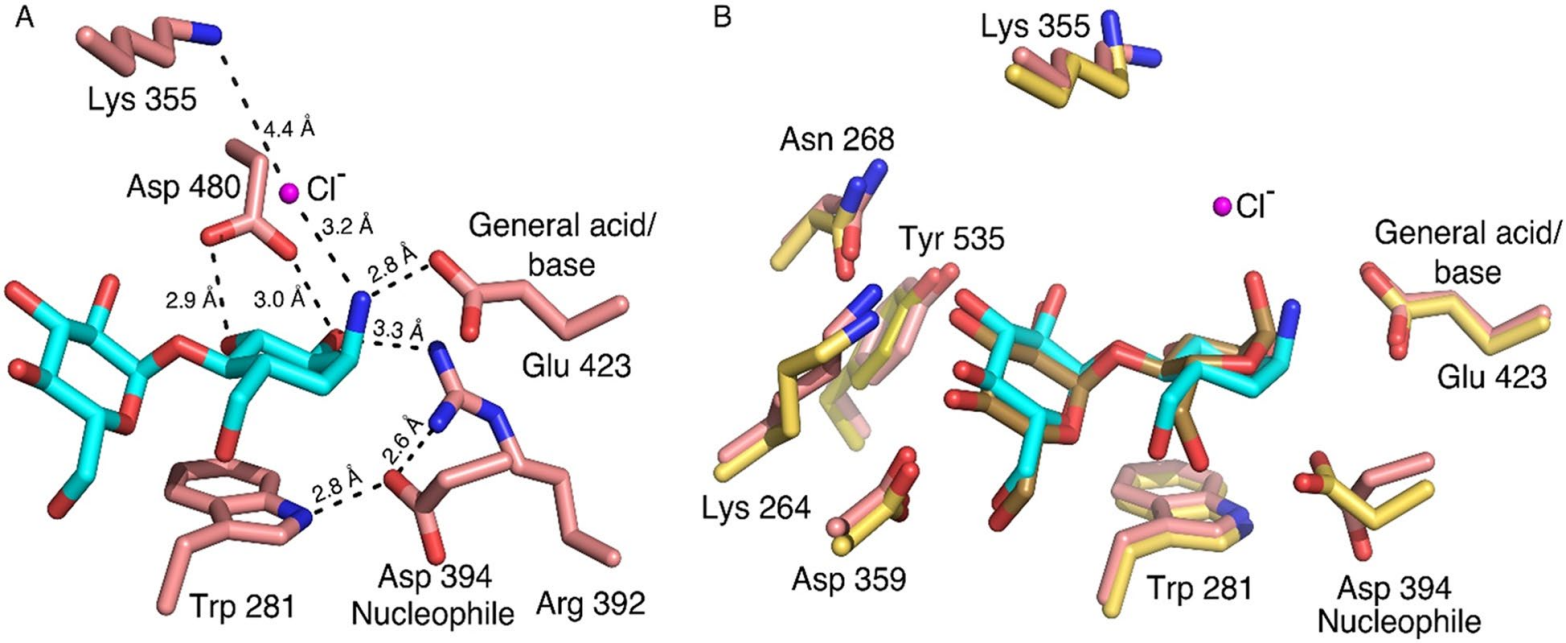
(**A**) The interactions formed in − 1 subsite of *Sco* GlgE1-V279S with **7**. (**B**) Superimposed active sites of *Sco* GlgE1-V279S/**7** and *Sco* GlgE1/Maltose structures (yellow carbon atoms).

In the *Sco* GlgE1-V279S/**7** complex, the interactions are similar to those observed in the substrate-bound (M1P and maltose) and substrate-mimic (MCP) complexes because the saturated carbocycle in **7** maintains the expected chair conformation. Specifically, the C(2)–OH and C(3)–OH functional groups form a bidentate hydrogen-bonded interaction with Asp 480, which is a unique feature to the GH13 family of proteins and has been observed previously in GlgE (Fig. 5A)^62,63^. In addition, the C(2)–OH forms a hydrogen bond with the sidechain nitrogen atom/NH1 of Arg 392.

**Figure 6:**
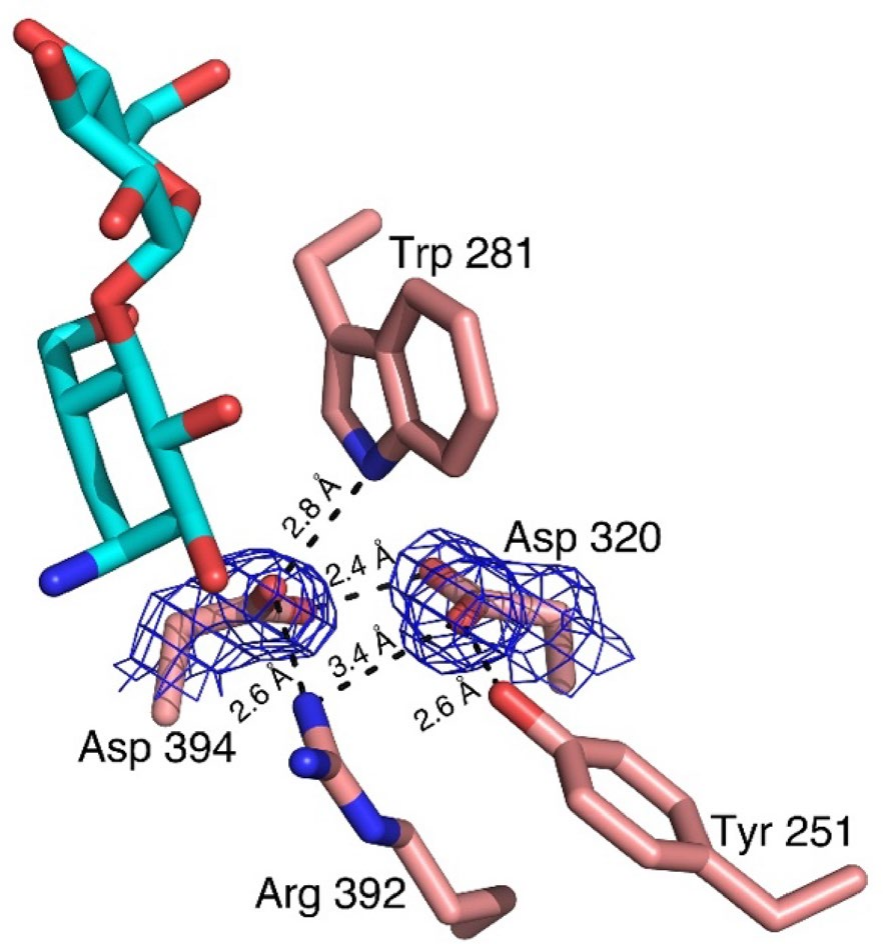
Interactions formed by shifted Asp 394 in *Sco* GlgE1-V279S/**7**, the composite 2F_O_ − F_C_ omit map was contoured at 1.5σ.

An unexpected difference between the *Sco* GlgE1-V279S/**7** complex structure and other complexes of disaccharide-like compounds is the rotation of the Asp 394 side chain away from the substrate analog. As this residue is known to form a hydrogen bond with the C6–OH group on incoming saccharides to allow positioning for nucleophilic attack on the anomeric carbon, it was anticipated to form the same hydrogen bond with **7**. Due to this bond rotation, Asp 394 in *Sco* GlgE1-V279S shifts away from the substrate binding site and does not form any direct interaction with **7** (Fig. 5A). The carboxylate moiety of Asp 394 is further stabilized by formation of hydrogen bonds with the side chain nitrogen atom/NH1 of Arg 392 and the indole of Trp 281. Additionally, the superimposed active sites of *Sco* GlgE1-V279S/**7** and *Sco* GlgE1/Maltose (PDB:3ZT5)^58^ complexes (Fig. 5B) clearly highlight χ^2^ dihedral rotation in Asp 394. While this structural change in Asp 394 is exhibited unambiguously by the 2F_O_ − F_C_ composite omit map density contoured at 1.5σ (Fig. 6), the reason for this movement of Asp 394 appears to derive from steric hindrance with atom C(1) of **7** apparently due to the attraction between the C(1) ammonium moiety and the Glu 423 side chain. Since the crystals were grown at pH 7.5, the Asp side chains would be expected to be in the carboxylate form and harbor a negative charge, it is likely that this attraction has shifted the carbocycle closer to Glu 423 than in other *Sco* GlgE1-V279S complexes. Inspection of the interaction network in this area illustrates that the side chains of Trp 281 and Tyr 251 form hydrogen bonds with each of the opposing aspartate side chains. Importantly, the Arg 392 side chain is positioned near the two aspartates and forms hydrogen bonds of 2.6 and 3.4 Å with Asp 394 and Asp 320, respectively. In apparent contrast to those stabilizing interactions, the 2.4 Å distance between O(D1) atoms of Asp 320 and Asp 394 represent the only complex-destabilizing interaction in this region. However, the very short distance between those atoms can be rationalized if either of the two side chains becomes protonated and the O(D1) atoms in those residues are using that hydrogen in a low barrier hydrogen bond. It is suspected that the steric hindrance producing this energetically unfavorable repositioning of Asp 394 significantly decreases the affinity of GlgE for compound **7**.

**Figure 7:**
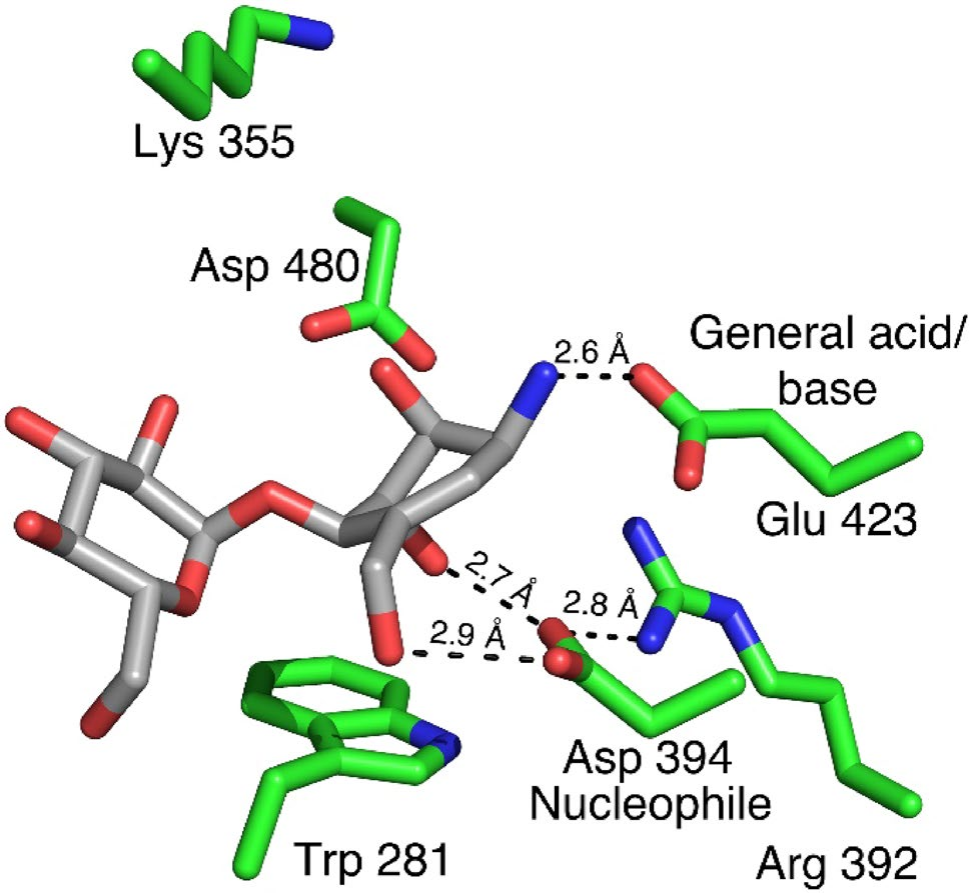
The interactions formed in the − 1 subsite of *Sco* GlgE1-V279S with **8**.

When inspecting the *Sco* GlgE1-V279S/**8** complex, the position of the amino acid residues forming the − 1 subsite do not undergo the structural change observed in the compound **7** complex as they are consistent with previous *Sco* GlgE1-V279S complexes. This can be explained by the structural differences observed in compound **8** resulting due to the presence of the *sp*^2^ hybridized carbons at positions 5 and 6 in the carbocycle. The ring exhibits a flattened conformation that differs significantly from the chair conformation in **7** (Fig. 7). This changes the hydroxyls at positions C(2) and C(3) from equatorial orientations in **7** to axial orientations in **8,** while the ammonium moiety on C1 takes a more equatorial orientation that positions the ammonium group within 2.6 Å of the Glu 423 side chain carboxylate. These significant structural differences in **8** mirror anticipated changes in the substrate during the enzymatic reaction. The C(3)–OH forms one of two hydrogen bonded interactions with the side chain of the Asp 394 nucleophile. The other hydrogen bond donor in this bidentate interaction is the C(7)–OH, whose location is nearly identical in both the **7** and **8** complex structures (Fig. 7). Indeed, all previously published structures of GlgE with any hexose analog have a hydroxyl at this position that interacts directly with the enzyme nucleophile^58,60^. Inspection of other GH13 enzymes in complex with Acarbose, which is a tetrasaccharide possessing an *N*-glycosidic linkage that functions as an inhibitor of α-amylases. Inspection of available GH13 enzymes in complex with Acarbose or Acarbose-derived oligosaccharides (accession codes: 4B9Z, 4E2O, 6GXV, and 6LGE) show that the *N*-glucosyl moieties in these structures maintain a chair conformation with C(2)–OH and C(3)–OH in equatorial positions^64–67^. The difference *Sco* GlgE1-V279S/**8** could be due to a low interconversion barrier for ^2^H_3_ to ^3^H_2_ in compound **8**.

The ammonium moieties in both compounds form polar interactions with the side chain of the general acid/base, Glu 423. This interaction is consistent with the role of Glu 423 in deprotonating the OH nucleophile during the second catalytic step and is likely stabilizing the complex due to the charge complementary between Glu 423 and the ammonium moieties. The difference in polar bond distances is a function of the ring flattening in **8** that affords closer approach of the ammonium to Glu 423 while avoiding steric hindrance with Asp 394 (Figs. 5A, 7). This shorter polar bond distance and lack of movement in Asp 394 may be the major factor defining the differences in affinity observed for **7** and **8**.

**Figure 8:**
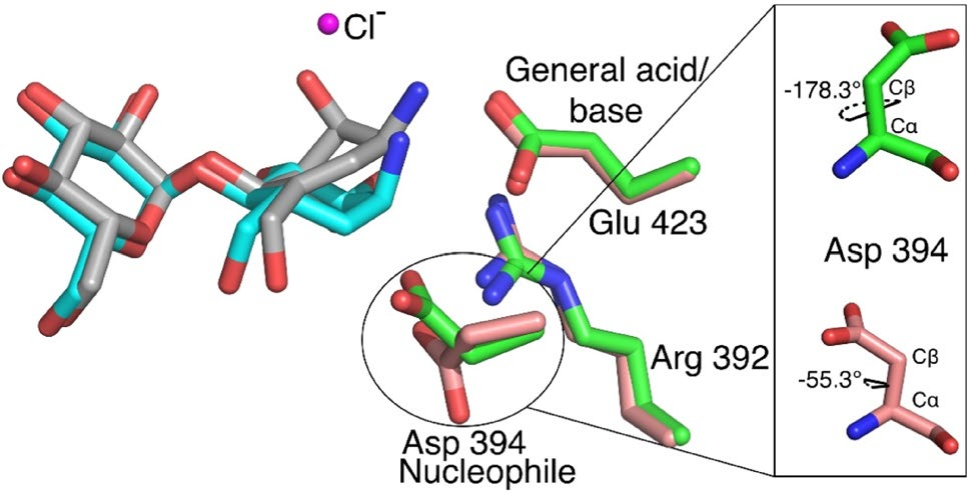
Superimposed active sites of *Sco* GlgE1-V279S/**7** and *Sco* GlgE1-V279S/**8** structures.

Further active site comparison of both structures (Fig. 8) indicates an upward shift in the carbocycle ring of compound **8**, as well as minor shifts in the side chains of Lys 355, Arg 392, Asp 394 and Glu 423, but these are likely within coordinate error. The superposition shown in Fig. 8 also highlights the significant rotation of the χ^2^ dihedral observed in the *Sco* GlgE1-V279S/**7** complex. When measuring the χ^1^ dihedral angle (between Cα and Cβ atoms) of Asp 394 in GlgE1-V279S/**8** and *Sco* GlgE1-V279S/**7**, we identified a 123° dihedral difference. Further, the superimposed active sites of Apo *Sco* GlgE1(PDB:3ZSS), *Sco* GlgE1-V279S/**7**, and *Sco* GlgE1-V279S/**8** complexes revealed the χ^2^ dihedral rotation in Asp 394 of *Sco* GlgE1-V279S/**7** complex (Supplementary Figure S1), and we noted a 119.6° dihedral difference between Asp 394 in Apo *Sco *GlgE1 and *Sco *GlgE1-V279S/**7**.

**Figure 9:**
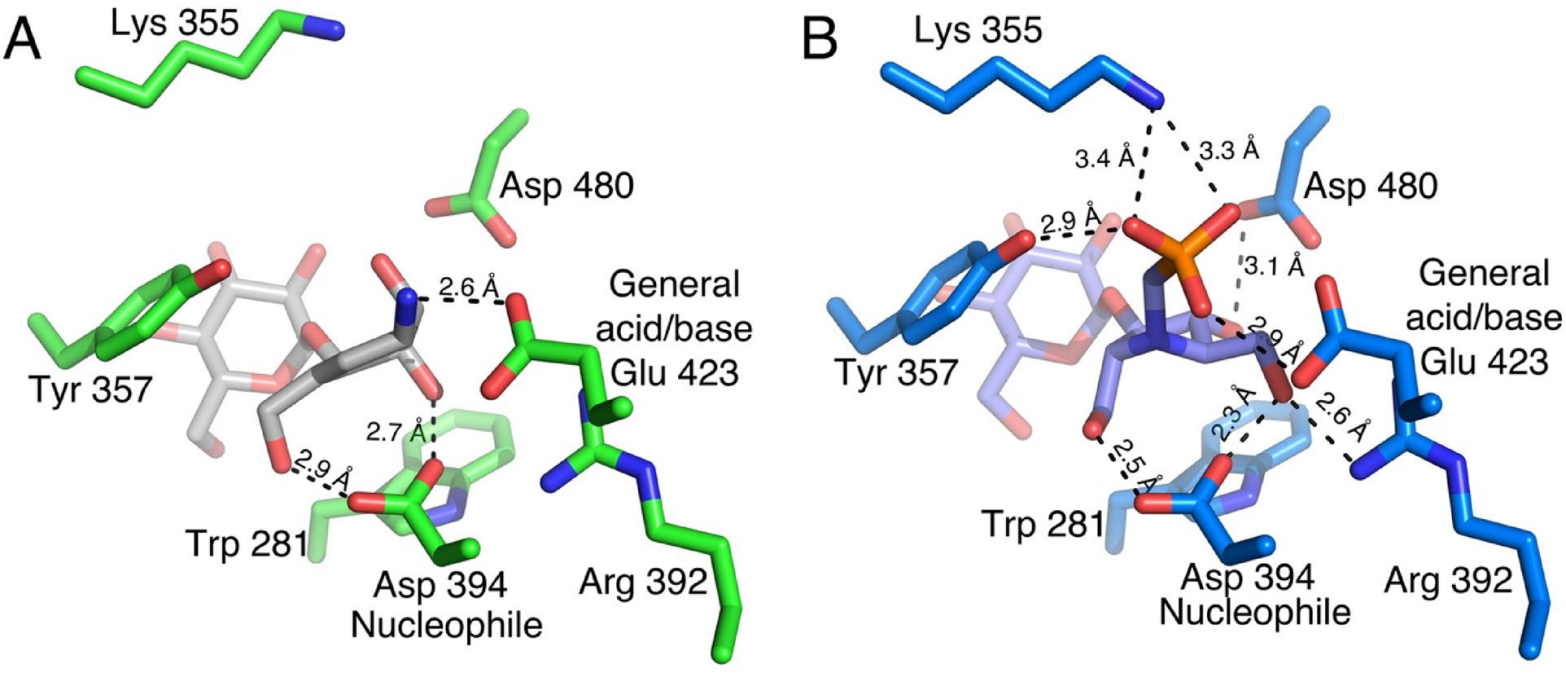
Comparison of interactions in the − 1 sites of (**A**) *Sco* GlgE1-V279S/**8** and (**B**) *Sco* GlgE1-V279S/MPP structures.

The previous studies relating to the synthesis and characterization of zwitterionic pyrrolidine-phosphonates showed that such compounds are reasonable mimics of the TSs formed during the enzymatic reaction. Out of those zwitterionic pyrrolidine-phosphonates, the methyl phosphonate pyrrolidine (MPP) is the most potent inhibitor against *Sco* GlgE1-V279S exhibiting an equilibrium dissociation constant (*K*_i_) of 45 ± 4 μM^60^. The comparison of the active sites (Fig. 9) of *Sco* GlgE1-V279S/**8** and *Sco* GlgE1-V279S/MPP (PDB:5VT4) clearly indicates interactions between GlgE residues forming the − 2 subsite and the glucose moiety bound within that site are conserved with respect to previously published GlgE structures. When comparing the − 1 subsite of both structures, the pyrrolidine ring of *Sco* GlgE1-V279S/MPP complex and the flattened carbocycle moiety of *Sco* GlgE1-V279S/**8** complex mimic some aspects of the enzyme substrate conformations adopted during the formation of the first TS. Specifically, the hydrogen bonded interactions that form between the C(7)–OH hydroxyl and OD1 of Asp 394 are identical for both *Sco* GlgE1-V279S/MPP and *Sco* GlgE1-V279S/**8** complexes. Additionally, the role of Glu 423 as both an acid and a base in the enzymatic reaction affords polar interactions in these two complexes with the side chain of Glu 423 forming a hydrogen bond with the negatively charged phosphonate in the MPP complex and a likely ionic interaction with the positively charged ammonium of compound **8**.

Major differences that significantly weaken binding of **8** to GlgE1-V279S relate to the loss of interactions with residues forming the phosphate binding site as well as hydrogen bonding interactions with the sugar mimetic in the − 1 site. As shown in Fig. 9B, Lys 355, Tyr 357, and Glu 423 form four interactions with the phosphomimetic in MPP. There is likely an ionic interaction between Asp 394 and the endocyclic nitrogen of MPP with a distance of 3.5 Å. Only one such interaction, the bond between Glu 423 and the ammonium moiety in **8**, is formed in the *Sco* GlgE1-V279S/**8** complex. Further, Arg 392 and Asp480 form hydrogen bonds with MPP and both of these interactions are lacking in the *Sco* GlgE1-V279S/**8** complex.

Further consideration of the *Sco* GlgE1-V279S/**8** and *Sco* GlgE1-V279S/MPP complexes suggest valuable information to design future inhibitors for *Sco* GlgE-V279S and the homologous *Mtb* GlgE. With this assessment, it seems that endocyclic amines are preferable to α-exocyclic amines because the approach of the positively charged group toward the Glu 423 side chain does not encroach upon the volume of space occupied by the Asp 394 side chain and therefore would not promote protein structural changes that are detrimental to inhibitor binding. Additionally, while GlgE can accommodate the axial hydroxyl moieties at positions 2 and 3, it seems that only the axial C(3)–OH group in **8** supports binding. Specifically, the benefit of an axial C(3)–OH is that it interacts directly with the Asp 394 side chain affording a bidentate interaction between the inhibitor and the enzyme nucleophile. In contrast, the axial C(2)–OH in **8** does not form any notable interactions with the enzyme. Indeed, the presence of an equatorial C(2)–OH group is beneficial for binding since it forms two hydrogen bonded interactions with amino acid residues in the − 1 subsite. Further, in the − 1 subsite of all the compound bound structures of *Sco* GlgE1-V279S, we observe some volume that can be targeted with additional functional groups. This includes the volume occupied by the phosphonate in MPP and the chloride ion in the *Sco* GlgE1-V279S/**7** complex. Therefore, we can extend the length of the new inhibitors to access those unoccupied areas and form additional interactions with the conserved amino acid residues present in those areas.

## Conclusions

The results obtained in these studies relate to both synthesis and structural evaluation of aminocarbocycles as inhibitors of GH13 enzymes. Herein, we have analyzed the diastereoselectivity of the Grignard reaction using the Cram’s-chelate TS model. It is noted that the selectivity follows the rule for 4-α-glucoside series. However, there is an erosion of stereoselectivity in the 4-α-glucoside series (4:1) with respect to the gluco series (9:1). We were successful in identifying the desired isomer **27A** after subjecting the obtained dienes (**27A** and **27B**) to RCM and comparing their spectral data with the alkenes **17A** and **17B**. The success of the synthetic route was highly dependent on the development of suitable experimental conditions due to highly sensitive nature of the Grubbs’ RCM. Also, we have successfully synthesized and determined the stereochemistry of the target 1-aminocarbasugars **7** and **8**. In the case of **7**, the stereoselectivity of the hydrogenation was improved relative to the gluco series. Of these two compounds, 1000 µM concentration of **8** was able to reduce the enzyme activity of *Sco* GlgE1-V279S by 65%. Compound **8** formed each of the expected interactions in the − 2 subsite as these are consistent with previously published *Sco* GlgE1-V279S structures. The origins of the inhibitory ability of **8** derives from the flattened ring system, coulombic interactions with conserved active site residues, and the ability to adopt a E_2_ conformation in the cyclohexene fragment. This causes the 3-OH to form a new hydrogen bond with the catalytic nucleophile while also maintaining an electrostatic interaction between the exocyclic amine and the general acid/general base Glu 423. The E_2_ conformation of **8** in the complex is likely a result of the favorable Glu 423—ammonium electrostatic interaction which would be absent in a ^2^H_3_ conformation and the low interconversion barrier for ^2^H_3_ to ^3^H_2_. Overall, the inclusion of the exocyclic amine was not as beneficial to binding as expected. While the short polar interactions between the amine and Glu 423 are likely stabilizing complex formation, this benefit is negated in the complex with compound **7** due to unexpected protein structural changes induced by steric hindrance. In conclusion, we have reported an effective regiospecific method to prepare the 4-α-glucosides of 1-aminocarbasugars using a readily available starting material. This structural and inhibitory information provides valuable insights into the atomic level understanding of valienamine-based GH inhibitor interactions and reveals new avenues for the design of improved inhibitors based on this important scaffold.

## Experimental methods

### General methods

All chemicals and solvents were purchased from Fisher Scientific, Acros Organics, Alfa Aesar or Sigma-Aldrich. Solvents were dried by through solvent purification system by passing through activated alumina and copper catalyst columns. All reactions were carried out at room temperature under a nitrogen atmosphere using a nitrogen balloon unless mentioned otherwise. Reactions were monitored by TLC (silica gel, f_254_) under UV light or by charring (5% H_2_SO_4_–MeOH) and the purification was performed by column chromatography on silica gel (230–400 mesh) using the solvent system specified, solvents were used without purification for chromatography. ^1^H NMR was recorded on Bruker Avance III 600 MHz spectrometer using CDCl_3_ and D_2_O as an internal reference. ^13^C were recorded on Bruker Avance III 600 MHz spectrometer using CDCl_3_ and D_2_O as internal reference. High resolution mass spectrometry was recorded on TOF MS-ES + instrument. Low resolution mass spectrometry was recorded on ESquire-LC–MS.

#### 1,2,3,6,2′ ,3′ ,4′ ,6′-octa-*O*-acetyl-D-maltose (21)^68^

D-(+)-Maltose monohydrate (2.00 g, 5.84 mmol) was suspended in dry pyridine (7.20 mL, 89.0 mmol) and acetic anhydride (6.28 mL, 66.8 mmol). A catalytic amount of 4-(dimethylamino)pyridine was added to the solution. The solution was stirred at ambient temperature for 16 h. The reaction mixture was diluted with ethyl acetate and washed successively with 1 N HCl (20 mL) and saturated aq. NaHCO_3_ (40 mL). The resulting organic phase was dried with anhydrous Na_2_SO_4_, filtered and the filtrate was concentrated under reduced pressure to give product **21**: yield 95% (3.50 g); silica gel TLC *R*_*f*_ = 0.5 (50% ethyl acetate: hexane). All the NMR values match with the reported data^68^.

#### 4-methylphenyl 1-thio-β-D-maltopyranopyranoside (22)^69^

Peracetylated maltose **21** (3.45 g, 5.08 mmol) was dissolved in dichloromethane. 4-Methylthiophenol (1.26 g, 10.2 mmol) and boron trifluoride diethyl etherate (0.75 mL, 6.10 mmol) were added to the solution at 0 °C. The resulting solution was stirred at ambient temperature under nitrogen atmosphere. The reaction was monitored by TLC and appeared to be complete after 4 h. The reaction mixture was diluted with dichloromethane (20 mL) and successively washed with saturated aq. NaHCO_3_ (25 mL), brine (25 mL), and water (15 mL). The resulting organic phase was dried (anhydrous Na_2_SO_4_), filtered and the filtrate was concentrated under reduced pressure. The product was dried overnight and deacetylated by dissolving in dry methanol followed by addition of a catalytic amount of sodium metal until the solution reached pH 9. The reaction was monitored for completion using TLC. The reaction was neutralized by adding Amberlite IRA-118H H^+^ resin until the pH reached 7. Then, the resin was filtered away and the filtrate was concentrated under reduced pressure to afford the 4-methylthiophenyl maltoside **22**: yield 90% (3.10 g). All the NMR values match with the reported data^69^.

#### 4-methylthiophenyl 2,3,6-Tri-***O***-benzyl-4-***O***-(2′,3′,4′,6′-tetra-***O***-benzyl-α-D-glucopyranosyl)-β-D-glucopyranoside (23)^70^

A solution of **22** (2.95 g, 9.04 mmol) in dry *N,N-*dimethylformamide (50 mL) was cooled to 0 °C. The solution was treated drop-wise with a suspension of sodium hydride (60% dispersion in mineral oil) (4.55 g, 114.0 mmol) in dry *N,N-*dimethylformamide. Benzyl bromide (11.6 mL, 95.3 mmol) was added drop-wise over 15 min. and the solution stirred at room temperature for 16 h. The reaction was poured over ice and extracted with ethyl acetate (50 mL). The combined organic layers were washed with brine. The resulting organic phase was dried (anhydrous Na_2_SO_4_), filtered and the filtrate was concentrated under reduced pressure to obtain a product. The product was purified by silica gel flash column chromatography. The product fractions were combined, concentrated, and dried in vacuum to afford a yellow oily product **23**: yield 85% (2.50 g); silica gel TLC *R*_*f*_ = 0.61 (25% ethyl acetate: hexane). All the NMR values match with the reported data^70^.

#### 2,3,6-Tri-***O***-benzyl-4-***O***-(2′,3′,4′,6′-tetra-O-benzyl-α-D-glucopyranosyl)-α/β-D-glucopyranoside (24)^52^

N-bromosuccinimide (0.90 g, 5.08 mmol) was added to a solution of **6** (2.47 g, 2.45 mmol) in 9:1 acetone–water (50 mL) and stirred at room temperature for 45 min. The solvent was evaporated at room temperature until turbid. A solution of the residue in ethyl acetate (100 mL) was washed successively with saturated aqueous NaHCO_3_, (3 × 50 mL) and water (3 × 50 mL). The solution was dried with anhydrous Na_2_SO_4_ and evaporated. The product was purified by silica gel flash column chromatography. The product fractions were combined, concentrated, and dried in vacuum to afford a yellow oil **24**: yield 97% (2.40 g); silica gel TLC *R*_*f*_ = 0.19 (30% ethyl acetate: hexane). All the NMR values match with the reported data^52^.

#### 3,4,7-Tri-***O***-benyl-5-***O***-(2′,3′,4′,6′-tetra-***O***-benzyl-α-D-glucopyranosyl)-D-gluchept-1-enitol (25)^34^

*n*-Butyl lithium (2.22 M) in hexanes (5.3 mL, 12.0 mmol) was added drop-wise to a suspension of methyltriphenylphosphonium bromide (3.36 g, 9.44 mmol) in tetrahydrofuran (50 mL) at − 20 °C. The solution was stirred at − 20 °C for 15 min. and raised to ambient temperature over 1 h. The solution was cooled to − 20 °C and compound **24** (2.30 g, 2.36 mmol) dissolved in 50 mL of tetrahydrofuran was added drop-wise. The solution was stirred at − 20 °C for 15 min. and allowed to warm to ambient temperature and stirred for an additional 6 h. The solution was diluted with acetone (6 mL) and stirred for 30 min. Diethyl ether (40 mL) was added to precipitate triphenylphosphine oxide. The latter was removed by filtration through Celite™ 545 filter aid. The filtrate was washed successively with satd. aq. NaHCO_3_ and brine (50 mL × 3). The solution was dried with anhydrous Na_2_SO_4_, filtered and the filtrate concentrated under reduced pressure to obtain the crude product. The product was purified by silica gel flash column chromatography on silica gel to give product as a yellow oil **25**: yield 55% (1.30 g); *R*_*f*_ = 0.63 (30% ethyl acetate : hexane). All the NMR values match with the reported data^34^_._

#### 3,4,7-Tri-***O***-benzyl-5-***O***-(2′,3′,4′,6′-tetra-***O***-benzyl-α-D-glucopyranosyl)-D-gluco-hept-1-enone (26)

Compound **25** (1.20 g, 1.24 mmol) was dissolved by gentle warming in anhydrous benzene (3.0 mL). To this solution was added dry dimethyl sulfoxide (3.0 mL). To the clear solution was added anhydrous pyridine (99.5 µL, 1.24 mmol), trifluoracetic acid (47.3 µL, 0.62 mmol) and *N,N*′-dicyclohexylcarbodiimide (0.76 g, 3.70 mmol) in that order. The reaction was left to stir at room temperature for 18 h. After completion of reaction, which was monitored by TLC, benzene (10 mL) was added. The resulting crystalline dicyclohexylurea was removed by filtration and washed with benzene. The combined filtrates and washings were extracted with water (20 mL × 3) to remove dimethyl sulfoxide. The organic layer was dried over anhydrous Na_2_SO_4_, evaporated under reduced pressure, and subjected to flash column chromatography on silica gel with 1:6 ethyl acetate–hexane to give product as colorless viscous liquid **26**: yield 88% (1.05 g); silica gel TLC *R*_*f*_ = 0.72 (30% ethyl acetate: hexane); ^1^H NMR (600 MHz, CDCl_3_) δ 7.33–7.23 (m, 33H, aromatic), 7.17–7.15 (m, 2H, aromatic), 5.97 (m, 1H, H-1), 5.24 (m, 2H, = C*H*_2_), 4.93 (d, *J* = 11 Hz, 1H, PhC*H*), 4.90–4.84 (m, 4H, PhC*H* , H-1′), 4.68 (dd, *J* = 12, 4.1 Hz, 2H, PhC*H*), 4.56 (dd, *J* = 12, 4.1 Hz, 2H, PhC*H*), 4.48 (d, *J* = 10.8 Hz, 1H, PhC*H*), 4.44–4.37 (m, 4H, PhC*H*, H-6a), 4,26 (d, *J* = 6.2 Hz, 1H, H-4), 4.18–4.24 (m, 3H, PhC*H*, H-6b), 4.09–4.06 (m, 2H, H-3′, H-2), 3.93 (m, 1H, H-5′), 3.85 (dd, *J* = 6.2, 4.4 Hz, 1H, H-3), 3.79–3.74 (m, 2H, H-4′, H-6a′), 3.54 (dd, *J* = 9.7, 3.7 Hz, 1H, H-2′), 3.45 (dd, *J* = 10.8, 1.8 Hz, 1H, H-6b′); ^13^C NMR (150 MHz, CDCl_3_): δ 204.40, 138.77, 138.53, 138.28, 138.19, 137.92, 137.91, 137.64, 134.92, 128.41, 128.34, 127.70, 127.63, 118.92, 99.72, 81.73, 80.64, 79.71, 79.71, 79.23, 75.64, 74.90, 74.55, 73.48, 73.20, 73.06, 70.74, 68.06 ppm; mass spectrum (HRMS), *m/z* = 969.4601 (M + H)^+^, C_62_H_64_O_10_ requires 969.4578.

#### 3,4,9-Tri-***O***-benzyl-5-***O***-(2′,3′,4′,6′-tetra-***O***-benzyl-α-D-glucopyranosyl)-D-gluco-octa-1,7-dienitol (27A)

To a cooled (− 78 °C) solution of **26** (1.04 g, 1.07 mmol) in tetrahydrofuran (15 mL) was added of vinylmagnesium bromide (0.7 M) in tetrahydrofuran (4.57 mL, 3.20 mmol) dropwise. The reaction mixture was stirred for 1 h. at the same temperature. The reaction mixture was warmed to room temperature. Diethyl ether (30 mL) and aq. NH_4_Cl (30 mL) were added to the reaction mixture. The organic layer was separated, washed with brine (50 mL × 2), and dried over anhydrous Na_2_SO_4_. The solvent was evaporated under reduced pressure and purification was performed by flash column chromatography on silica gel with 1:8 ethyl acetate–hexane to afford product as colorless viscous liquid **27A**: yield 93% (0.97 g); silica gel TLC *R*_*f*_ = 0.65 (30% ethyl acetate : hexane); ^1^H NMR (600 MHz, CDCl_3_) δ 7.40–7.23 (m, 31H, aromatic), 7.22–7.17 (m, 4H, aromatic), 6.30 (dd, *J* = 17.4, 10.6 Hz, 1H, H-7), 5.79 (ddd, *J* = 17.6, 10.4, 7.5 Hz, 1H, H-2), 5.59 (dd, *J* = 17.4, 1.7 Hz, 1H, H-8a), 5.37 (br dd, *J* = 17.3, 1.1 Hz, 1H, H-1a), 5.33 (d, *J* = 3.4 Hz, 1H, H-1′), 5.27 (ddd, *J* = 10.2, 6.8, 1.7 Hz, 2H, H-1b, H-8b), 5.01 (d, *J* = 10.9, 10.9 Hz, 2H, PhC*H*), 4.89 (d, *J* = 11.5 Hz, 2H, PhC*H*), 4.66–4.43 (m, 10H, PhC*H*, H-3), 4.29 (d, *J* = 11.7 Hz, 1H, PhC*H*), 4.11–4.03 (m, 2H, H-5, H-3′), 4.00–3.92 (m, 2H, H-5′, H-4), 3.85 (brs, 1H, OH), 3.76–3.68 (m, 2H, H-4′, H-9a), 3.64 (dd, *J* = 9.8, 3.5 Hz, 1H, H-2′), 3.55 (dd, *J* = 10.8, 2.8 Hz, 1H, H-6a′), 3.44 (dd, *J* = 10.7, 1.6 Hz, 1H, H-6b′), 3.36 (d, *J* = 8.9 Hz, 1H, H-9b). ^13^C NMR (150 MHz, CDCl_3_): δ 139.92, 138.86, 138.71, 138.60, 1338.38, 137.99, 137.97, 137.71, 135.78, 128.35, 128.30, 128.18, 128.11, 127.92, 127.75, 127.65, 127.58, 127.48, 127.43, 127.35, 127.24, 119.35, 1155.67, 97.01, 82.97, 81.77, 79.74, 79.37, 77.61, 77.49, 75.47, 74.93, 74.78, 74.32, 73.44, 73.02, 72.54, 70.77, 70.48, 67.81 ppm; mass spectrum (HRMS), *m/z* = 997.4899 (M + H)^+^, C_64_H_68_O_10_ requires 997.4891.

#### 3,4,9-Tri-***O***-benzyl-5-***O***-(2′,3′,4′,6′-tetra-***O***-benzyl-α-D-glucopyranosyl)-L-ido-octa-1,7-dienitol (27B)

Flash column chromatography on silica gel with 1:6 ethyl acetate–hexane afforded product as colorless liquid **27B**: yield 23% (0.24 g); silica gel TLC *R*_*f*_ = 0.72 (30% ethyl acetate : hexane); ^1^H NMR (600 MHz, CDCl_3_) δ 7.35–7.13 (m, 33H, aromatic), 7.04 (m, 2H, aromatic), 6.27 (dd, *J* = 17.3, 10.9 Hz, 1H, H-7), 5.81 (ddd, *J* = 17.3, 10.2, 8.3 Hz, 1H, H-2), 5.60 (dd, *J* = 17.4, 1.9 Hz, 1H, H-8a), 5.44 (d, *J* = 3.4 Hz, 1H, H-1′), 5.37 – 5.26 (m, 3H, H-1a, H-1b, H-8b), 4.98 (d, *J* = 10.8 Hz, 1H, PhC*H*), 0.4.93 (d, *J* = 11.0 Hz, 1H, PhC*H*), 0.4.84 (d, *J* = 10.8 Hz, 1H, PhC*H*), 4.80 (d, *J* = 10.8 Hz, 1H, PhC*H*), 4.60 (dd, *J* = 16.5, 11.3 Hz, 2H, PhC*H*), 4.49–4.33 (m, 8 H, PhCH), 4.25 (t, *J* = 8.3, 8.3 Hz, 1H, H-3), 4.05 (d, *J* = 1.8 Hz, 1H, H-5), 3.95–3.90 (m, 2H, H-4, H-3′), 3.84 (d, *J* = 9.2 Hz, 1H, H-9a), 3.77 (m, 1H, H-5′), 3.69–3.63 (m, 1H, H-4′), 3.54 (dd, *J* = 9.3, 4.6 Hz, 1H, H-2′), 3.39 (dd, *J* = 10.6, 2.7 Hz, 1H, H-6a′), 3.33–3.29 (m, 2 H, H-6b′, H-9b), 2.97 (brs, 1H, OH); ^13^C NMR (150 MHz, CDCl_3_): δ 139.20, 138.81, 138.68, 138.59, 138.35, 137.93, 137.77, 137.20, 135.86, 128.28, 127.63, 127.54, 127.13, 120.06, 115.69, 94.33, 84.60, 81.93, 79.01, 77.90, 77.30, 78.15, 75.46, 74.46, 74.86, 74.67, 73.43, 72.78, 71.15, 70.44, 67.86 ppm; mass spectrum (HRMS), *m/z* = 997.4899 (M + H)^+^, C_64_H_68_O_10_ requires 997.4891.

#### (***1***D)-(1,3,4/2)-1,2-Di-***O***-benzyl-4-***C***-[(benzyloxy)methyl]-3-***O***-(2′,3′,4′,6′-tetra-***O***-benzyl-α-D-glucopyranosyl)cyclohex-5-ene-1,2,3,4-tetrol (28A)

A solution of dialkene **27A** (0.95 g, 0.95 mmol) in dry dichloromethane (25 mL) was degassed by passing nitrogen gas through it for 20 min. After that, 1st generation Grubbs’ catalyst (10 mol%) was added to the solution and the reaction was kept under reflux conditions in nitrogen atmosphere for 5 days or until the catalyst turned dark brown. The catalyst was removed by filtration through Celite™ 545 filter aid. The filtrate was evaporated under reduced pressure and purified using flash column chromatography on silica gel to obtain yellowish liquid **28A**: yield 25% (0.25 g); silica gel TLC *R*_*f*_ = 0.5 (30% ethyl acetate : hexane); ^1^H NMR (600 MHz, CDCl_3_) δ 7.30–7.20 (m, 33H, aromatic), 7.19–7.11 (m, 2H, aromatic), 5.96 (dd, *J* = 10.2, 1.9 Hz, 1H, H-6), 5.69 (dd, *J* = 10.3, 1.8 Hz, 1H, H-5), 5.63 (d, *J* = 3.7 Hz, 1H, H-1′), 4.98 (d, *J* = 11.7 Hz, 1H, PhC*H*), 4.91–4.81 (m, 3H, PhC*H*), 4.77 (d, *J* = 10.9 Hz, 1H, PhC*H*), 4.67 (dd, *J* = 29.4, 11.7 Hz, 2H, PhC*H*), 4.59–4.43 (m, 6H, PhC*H*), 4.36 (d, *J* = 12.2 Hz, 1H, PhC*H*), 4.22 (m, 1H, H-1), 4.21–4.17 (m, 2H, H-2, H-3), 3.97 (t, *J* = 9.3 Hz, 1H, H-3′), 3.89 (m, 1H, H-5′), 3.70–3.63 (m, 2H, H-4′, H-7b), 3.56 (ddd, *J* = 13.5, 10.2, 3.5 Hz, 2H, H-2′, H-6a′), 3.49(dd, *J* = 10.5, 1.8 Hz, 1H, H-6b′), 3.39 (d, *J* = 9.1 Hz, 1H, H-7a), 3.22 (brs, 1H, OH); ^13^C NMR (150 MHz, CDCl_3_): δ 139.10, 138.66, 138.34, 138.31, 138.10, 137.96, 137.78, 130.75, 130.31, 128.35, 128.33, 128.27, 128.17, 128.08, 127.92, 127.84, 127.77, 127.65, 127.55, 127.41, 127.66, 126.92, 97.02, 81.77, 80.82, 80.10, 79.60, 77.54, 75.48, 74.95, 74.12, 73.49, 73.10, 72.98. 72.74, 71.50, 68.10 ppm; mass spectrum (HRMS), *m/z* = 991.4418 (M + Na)^+^, C_62_H_64_O_10_ requires 991.4397.

#### (***1***D)-(1,3,4/2)-1,2-Di-***O***-benzyl-4-***C***-[(benzyloxy)methyl]-3-***O***-(2′,3′,4′,6′-tetra-***O***-benzyl-α-D-glucopyranosyl)cyclohex-5-ene-1,2,3,4-tetrol (28B)

A solution of dialkene **27B** (0.22 g, 0.22 mmol) in dry dichloromethane (10 mL) was degassed by passing nitrogen gas through it for 20 min. After that, 1st generation Grubbs’ catalyst (10 mol%) was added to the solution and the reaction was kept under reflux conditions in nitrogen atmosphere for 5 days or until the catalyst turned dark brown. The catalyst was removed by filtration through Celite™ 545 filter aid. The filtrate was evaporated under reduced pressure and purified using flash column chromatography on silica gel to obtain yellowish liquid **28B**: yield 25% (60.0 mg); silica gel TLC *R*_*f*_ = 0.5 (30% ethyl acetate : hexane); ^1^H NMR (600 MHz, CDCl_3_) δ 7.35–7.15 (m, 35H, aromatic), 5.74 (ddd, *J* = 22.2, 10.4, 1.9 Hz, 2H, H-5, H-6), 5.32 (d, *J* = 3.8 Hz, 1H, H-1′), 5.04 (d, *J* = 12.0 Hz, 1H, PhC*H*), 4.93 (d, *J* = 10.9 Hz, 1H, PhC*H*), 4.87–4.76 (m, 3H, PhC*H*), 4.42–4.64 (m, 9H, PhC*H*), 4.27 (dt, *J* = 7.0, 1.8 Hz, 1H, H-1), 4.16 (dd, *J* = 10.5, 7.1 Hz, 1H, H-2), 3.95–3.89 (m, 2H, H-3, H-3′), 4.93 (d, *J* = 10.9 Hz, 1H, PhC*H*), 4.93 (d, *J* = 10.9 Hz, 1H, PhC*H*), 3.73 (d, *J* = 9.4 Hz, 1H, H-7a), 3.67 (d, *J* = 9.4 Hz, 1H, H-7b), 3.61–3.51 (m, 4H, H-2′, H-4′, H-6a′, H-6b′), 3.40 (brs, 1H, OH); ^13^C NMR (150 MHz, CDCl_3_): δ 139.37, 138.72, 136.64, 136.21, 138.13, 137.99, 137.80, 132,14, 128.41, 128.38, 128.37, 128.32, 128.17, 128.10, 128.06, 128.03, 127.90, 127.80,, 127.76, 127.73, 127.62, 127.56, 127.47, 126.99, 126.95, 126.66, 99.37, 84.31, 81.67, 80.95, 80.46, 79.24, 77.24, 75.52, 75.12, 75.05, 74.37, 73.62, 73.58, 73.53, 72.41, 71.33, 71.11, 68.51, 29.73 ppm; mass spectrum (HRMS), *m/z* = 991.396 (M + Na)^+^, C_62_H_64_O_10_ requires 991.4397.

#### (***1***D)-(1,3,4/2)-1,2-Di-***O***-benzyl-4-***C***-[(benzyloxy)methyl]-4-***O***-carbamoyl-3-***O***-(2′,3′,4′,6′-tetra-***O***-benzyl-α-D-glucopyranosyl)cyclohex-5-ene-1,2,3,4-tetrol (29)

A solution of **28A** (0.24 g, 0.25 mmol) in dichloromethane (10 mL) was cooled at 0 °C and treated dropwise with trichloroacetyl isocyanate (60 µL, 0.50 mmol). The reaction mixture was stirred for 30 min. at the same temperature and evaporated. The residue was dissolved in methanol (10 ml) and water (1 ml), was cooled to 0 °C and treated with potassium carbonate (0.07 g, 0.49 mmol). The reaction mixture was stirred for 2 h. at that temperature and warmed to room temperature and again stirred for another 2 h. After completion of the reaction, methanol was evaporated and the aq. solution. was diluted with water (10 mL) and extracted with dichloromethane (20 mL × 3). The organic layer was separated washed with brine (10 mL × 3), dried over anhydrous Na_2_SO_4_. The solvent was evaporated under reduced pressure and purification was performed by flash column chromatography on silica gel with with 1:3 ethyl acetate–hexane to afford product as colorless liquid **29**: yield 85% (0.21 g); silica gel TLC *R*_*f*_ = 0.25 (30% ethyl acetate : hexane); ^1^H NMR (600 MHz, CDCl_3_) δ 7.36–7.20 (m, 33H, aromatic), 7.16–7.14 (m, 2H, aromatic), 6.32 (dd, *J* = 10.2, 1.9 Hz, 1H, H-6), 6.01 (dd, *J* = 10.3, 1.8 Hz, 1H, H-5), 5.53 (d, *J* = 3.7 Hz, 1H, H-1′), 5.07 (d, *J* = 11.7 Hz, 1H, PhC*H*), 4.92 (m, 2H, PhC*H*), 4.86 (d, *J* = 11.0 Hz, 1H, PhC*H*), 4.65 (dd, *J* = 11.4, 9.0 Hz, 2H, PhC*H*), 4.59–4.56 (m, 2H, PhC*H*), 4.52–4.45 (m, 4H, 2 PhC*H*), 4.35 (d, *J* = 12.0 Hz, 1H, PhC*H*) 4.28–4.23 (m, 3H, H-1, H-2, H-3), 4.14–4.08 (m, 3H, H-3′, H-7a, H-7b), 4.02 (m, 1H, H-5′), 3.68 (t, *J* = 9.6, 9.6 Hz, 1H, H-4′), 3.61 (dd, *J* = 10.2, 3.0 Hz, 1H, H-6a′), 3.54 (dd, *J* = 9.6, 3.6 Hz, 1H, H-2′), 3.50 (d, *J* = 10.2 Hz, 1H, H-6b′). ^13^C NMR (150 MHz, CDCl_3_): δ 155.39, 139.32, 138.84, 138.30, 138.24, 138.17, 138.13, 137.87, 130.68, 129.67, 128.41, 128.39, 128.36, 128.31, 128.25, 128.19, 128.12, 128.05, 127.99, 127.93, 127.90, 127.77, 127.69, 127.57, 127.53, 127.39, 127.07, 126.97, 98.13, 81.53, 81.09, 80.50, 79.77, 79.75, 77.77, 75.93, 75.47, 75.16, 74.43, 73.47, 73.16, 72.50. 71.76, 70.90, 69.78, 68.51 ppm; mass spectrum (HRMS), *m/z* = 1034.4463 (M + Na)^+^, C_63_H_65_NO_11_ requires 1034.4455.

#### (***1***D)-(1,3,4/2)-1,2-Di-***O***-benzyl-6-[(benzyloxycarbonyl) amino]-4-[(benzyloxy)methyl]-3-***O***-(2′,3′,4′,6′-tetra-***O***-benzyl-α-D-glucopyranosyl) cyclohex-4-ene-1,2,3-triol (30)

Trifluoracetic anhydride (56.0 µL, 0.39 mmol) was added to a solution of carbamate **29** (0.20 g, 0.19 mmol) and triethylamine (165 µL, 1.19 mmol) in dry tetrahydrofuran (10 mL) and was cooled to 0 °C. The resulting mixture was slowly warmed to room temperature and stirred for 1 h. In a separate flask, sodium hydride suspension in 60% mineral oil (0.01 g, 0.39 mmol) was added to a solution of benzyl alcohol (41.0 µL, 0.39 mmol) in dry tetrahydrofuran (2 mL) at 0 °C. After 1 h, the solution of sodium benzyloxide was added to the generated isocyanate and the reaction progress was followed by TLC. After 24 h. volatiles are removed under reduced pressure. The reaction mixture was diluted with water (10 mL) and extracted with dichloromethane (10 mL × 4). The organic layer was separated washed with brine (10 mL × 2), dried over anhydrous Na_2_SO_4_. The solvent was evaporated under reduced pressure and purification was performed by flash column chromatography on silica gel with 1:5 ethyl acetate–hexane to afford product as colorless liquid **30**: yield 60% (0.13 g); silica gel TLC *R*_*f*_ = 0.43 (30% ethyl acetate : hexane); ^1^H NMR (600 MHz, CDCl_3_) δ 7.23–7.11 (m, 40H, aromatic), 5.92 (d, *J* = 3.0 Hz, 1H, H-5), 5.49 (d, *J* = 9.7 Hz, 1H, N*H*), 5.10–5.05 (m, 3H, H-1′, cbz), 4.79–4.71 (m, 3H, H-6, PhC*H*), 4.68–4.57 (m, 6H, PhC*H*), 4.51–4.36 (m, 6H, PhC*H*), 4.29 (br d, *J* = 4.8 Hz, 1H, H-3), 4.25 (d, *J* = 12.6 Hz, 1H, H-7a), 4.04 (dd, *J* = 4.8, 1.9 Hz, 1H, H-2), 3.94 (d, *J* = 12.6 Hz, 1H, H-7b), 3.90–3.87 (m, 2H, H-3′, H-5′), 3.76 (t, *J* = 4.8, 4.8 Hz, 1H, H-1), 3.68 (t, *J* = 9.6 Hz, 1H, H-4′), 3.63 (dd, *J* = 10.6, 2.9 Hz, 1H, H-6a′), 3.56 (dd, *J* = 9.8, 3.6 Hz, 1H, H-2′), 3.47 (dd, *J* = 10.6, 1.9 Hz, 1H, H-6b′). ^13^C NMR (150 MHz, CDCl_3_): δ 156.23, 138.71, 138.41, 138.23, 138.01, 137.93, 137.92, 136.65, 135.29, 128.49, 128.46, 128.41, 128.36, 128.34, 128.28, 127.99, 127.98, 127.92, 127.86, 127.74, 127.69, 127.59, 127.52, 127.49, 127.35, 97.58, 81.98, 79.52, 77.67, 75.93, 75.61, 75.33, 74.91, 73.96, 73.49, 73.03, 71.81, 71.77, 71.26, 71.03, 70.95, 68.16, 66.61, 46.54 ppm; mass spectrum (HRMS), *m/z* = 1124.4922 (M + Na)^+^, C_70_H_71_NO_11_ requires 1124.4925.

#### ***L***-chiro-Inisitol-1-amino-1,5,6-trideoxy-4-***O***-(α-D-glucopyranosyl)-5-(hydroxymethyl) or 4-***α***-glycoside derivative of validamine (7)

Compound **30** (40.0 mg, 0.04 mmol) was dissolved in 10 mL of methanol and 5 wt. % Pd/C (20.0 mg) was added. The mixture was stirred for 16 h. under hydrogen (1 atm.). The catalyst was filtered away through a plug of Celite® 545 and washed with methanol (5 mL). The combined filtrate and washings were concentrated to dryness and the residue was passed through reverse phase C-18 silica gel (washed with water) to afford **7** as a white solid: yield quantitative (5.00 mg); ^1^H NMR (600 MHz, D_2_O) δ 5.09 (d, *J* = 3.84 Hz, 1 H), 4.21 (m, 1 H), 3.92 (m, 2 H), 3.75–3.52 (m, 8 H), 3.35–3.32 (m, 1H), 2.13 (m, 1H), 1.71–1.55 (m, 2H). ^13^C NMR (151 MHz, D_2_O): δ 96.30, 73.06, 72.87, 72.69, 70.93, 69.04 (2C), 66.14, 62.37, 60.25, 48.94, 36.62, 21.63 ppm; mass spectrum (HRMS), *m/z* = 340.1599 (M + H)^+^, C_13_H_25_NO_9_ requires 340.1600.

#### α-D-Glucopyranoside, 4-amino-5,6-dihydroxy-2-(hydroxymethyl)-2-cyclohexen-1-yl or 4-***α***-glycoside derivative of valienamine (8)

Ammonia was condensed into a solution of **30** (60.0 mg, 0.05 mmol) in tetrahydrofuran (5 ml) using a dry ice cooled cold finger apparatus. The solution was treated with sodium in small pieces, until a blue color in the solution persisted. After stirring for 10 min. at − 78 °C temperature the mixture was treated with NH_4_Cl (100 mg), stirred at room temperature overnight and evaporated. The residue was extracted with methanol, filtered, and evaporated. The residue (20.0 mg) was adsorbed on 3 mL of neutral *Dowex 50-WX-8* (washed with water). After washing with water (10 mL), elution with 2% aqueous ammonia gave **8** as a white solid: yield quantitative (5.00 mg); ^1^H NMR (600 MHz, D_2_O): δ 5.71 (d, *J* = 4.2 Hz, 1H), 5.26 (d, *J* = 3.9 Hz, 1H), 4.16–4.06 (m, 3H), 3.93–3.85 (m, 3H), 3.71–3.58 (m, 3H), 3.50–3.40 (m, 2H), 3.27–3.23 (m, 1H). ^13^C NMR (151 MHz, D_2_O): δ 142.85, 117.37, 97.76, 75.27, 72.65, 72.63, 71.09, 70.14, 69.17, 66.40, 61.34, 60.31, 48.57 ppm; mass spectrum (HRMS), *m/z* = 338.1440 (M + H)^+^, C_13_H_23_NO_9_ requires 338.1446.

### Protein purification

The expression construct of *Sco* GlgE1-V279S (pET32-*Sco* GlgE1-V279S)^34^, that encodes for a non-cleavable C-terminal polyhistidine tagged protein, was used to transform T7 express E. coli cells. The large-scale cultures were grown at 37 °C in LB media with 264 mM concentration of carbenicillin. When O.D_600nm_ reached 0.6, cultures were cooled to 16 °C and cells were induced by the addition of 1 mM Isopropyl β-D-1-thiogalactopyranoside. After 16 h of induction, cells were harvested by centrifugation and resuspended in a lysis buffer consisting of 20 mM Tris pH 7.5, 500 mM NaCl, 10% glycerol, 0.5 mM imidazole and 0.3 mM tris(2-carboxyethyl) phosphine (TECP). The cell suspension was incubated on ice with lysozyme and DNase I for 30 min and lysed by sonication. The resulting lysate was clarified by centrifugation and subjected to a 5 mL metal affinity cobalt column that had been equilibrated with lysis buffer. The unbound protein was washed by passing 25 column volumes of lysis buffer through the column and *Sco* GlgE1-V279S was eluted isocratically with the elution buffer of 20 mM Tris pH 7.5, 500 mM NaCl, 150 mM imidazole and 0.3 mM TCEP. The excess salt and imidazole were removed from *Sco* GlgE1-V279S by overnight dialysis against a buffer consisting of 20 mM Tris pH 7.5, 150 mM NaCl and 0.3 mM TCEP.

### Inhibition studies

The inhibition studies for **7** and **8** were performed separately by using EnzChek Phosphate Assay Kit. Prior to the assay, stock solutions of 1 mM MESG, 20 mM concentrations of **7** and **8** (dissolved dH_2_O) were prepared and stored at − 20 °C. In the assay, all the reactions were carried out in a 96-well format, 50 µL reaction volume at 25 °C for 20 min in a continuous manner. The reactions were initiated by addition of 50 nM *Sco* GlgE1-V279S to the reaction mixture consisting of 20 mM Tris pH 7.5, 150 mM NaCl, 500 nM MESG, 0.25 U PNP, 0.5 mM glycogen (as maltose acceptor) and 250 µM M1P^34,60^. In the various reactions, the compound **8** concentration was varied from 0 to 1000 µM, except in the positive control that lacked inhibitor, and the negative control that lacked enzyme. All of the reactions were performed in triplicate using a Synergy H4 plate reader (Bio Tek) to measure the absorbance at 360 nm. The percent enzymatic activity was calculated by using the equation (V/V_0_) × 100, where V and V_0_ denote the rates of the inhibited and uninhibited enzyme (positive control), respectively. Using this assay, 100 µM concentration of both **7** and **8** were tested separately against *Sco* GlgE1-V279S. Dose–response experiments for **8** were performed as described above while varying the concentration of **8**.

### Crystallization of *Sco* GlgE1-V279S complexes

Prior to the experiment, 20 mM aqueous solutions of **7** and **8** were prepared. The protein-compound mixture was prepared by mixing the *Sco* GlgE1-V279S with each compound separately to a final concentration as 8 mg/mL^59^ of *Sco* GlgE1-V279S and 10 mM of the respective compound. The hanging drop vapor diffusion method was used for the crystallization and each crystallization drop consisted with 2 µL of *Sco* GlgE1-V279S/compound mixture and 2 µL of the well solution (0.2 mM Sodium citrate pH 7 and 10% PEG 3,350)^53^. The crystallization drop was equilibrated against 100 µL of the well solution and crystals were formed within 3 days. Following crystal formation, an additional 4 µL of the appropriate 20 mM compound stock was added to the drop and incubated for an additional 12 h. This results in a compound concentration of at least 15 mM in each crystallization drop, and would be nominally higher when considering drop dehydration from vapor diffusion. Crystals were cryoprotected by adding 4 µL of 50% PEG 2000 to the crystallization drop immediately prior to flash-cooling by immersion in liquid nitrogen in preparation for the cryo-crystallographic diffraction experiments.

### X-ray diffraction experiments

The LS-CAT beamline at Advanced Photon Source of Argonne National Labs, IL was used to perform the X-ray diffraction experiments. The X-ray diffraction data corresponding to the *Sco* GlgE1-V279S/**7** and *Sco* GlgE1-V279S/**8** complexes were indexed and reduced, using DIALS, iMosflm, and Scala in the CCP4 suite. The refinements were carried out by using PHENIX, and the previously published *Sco* GlgE1-V279S in complex with maltose-C-phosphonate (RCSB accession number 4U31) was used for difference Fourier analysis to calculate phases and difference maps prior to manual and computational refinement^71^. The visualization and manual refinements of the structures were carried out using COOT^72^. The final occupancy of each ligand was changed manually and then refined in PHENIX.

## Supplementary Information

Supplementary Information.
