# Author Correction: Stereoselective synthesis of a 4-⍺-glucoside of valienamine and its X-ray structure in complex with *Streptomyces coelicolor* GlgE1-V279S

**DOI:** 10.1038/s41598-021-96573-4

**Published:** 2021-08-18

**Authors:** Anshupriya Si, Thilina D. Jayasinghe, Radhika Thanvi, Sunayana Kapil, Donald R. Ronning, Steven J. Sucheck

**Affiliations:** 1 Department of Chemistry & Biochemistry, University of Toledo, Toledo, OH 43606 USA; 2 Department of Pharmaceutical Sciences, University of Nebraska Medical Center, Omaha, NE 68198 USA; 3 Merck Through ExecuPharm, Rahway, NJ 07065 USA

Correction to: *Scientific Reports* 10.1038/s41598-021-92554-9, published online 28 June 2021

Sunayana Kapil was omitted from the author list in the original version of this Article.

The Author Contributions section now reads:

A.S. prepared compounds **7** and **8**, S.K. prepared compounds **28A** and **28B**, and R.T. prepared all other chemical intermediates. T.D.J. completed kinetics and X-ray studies. All authors wrote their respective portions of the main manuscript text, supporting information text, and figures. D.D.R. and S.J.S. conceived and directed the structural biology and chemistry, respectively, and edited the manuscript. All authors reviewed the manuscript.

The original Article has been corrected.

