# Supplementary material for: Stereoselective synthesis of a 4-⍺-glucoside of valienamine and its X-ray structure in complex with Streptomyces coelicolor GlgE1-V279S
Source: Sci Rep. 2021 Jun 28;11:13413. doi: 10.1038/s41598-021-92554-9 (PMC8238978; doi:10.1038/s41598-021-92554-9)
Supplement: Supplementary file 1 — Supplementary Information. [file 41598_2021_92554_MOESM1_ESM.docx]

**Supporting Information**

**for**

Stereoselective synthesis of a 4-⍺-glucoside of valienamine and its X-ray structure in complex with *Streptomyces coelicolor* GlgE1-V279S

Anshupriya Si^‡1^, Thilina D. Jayasinghe^‡2^, Radhika Thanvi^1^, Sunayana Kapil^3^, Donald R. Ronning^2^*, Steven J. Sucheck^1^*

^1^ Department of Chemistry & Biochemistry, University of Toledo, Toledo, OH 43606, United States.

^2^ Department of Pharmaceutical Sciences, University of Nebraska Medical Center, Omaha, NE 68198

^3^ Merck through ExecuPharm, Rahway, NJ 07065

* Correspondence: [don.ronning@unmc.edu](mailto:don.ronning@unmc.edu); [steve.sucheck@utoledo.edu](mailto:steve.sucheck@utoledo.edu)

‡ These authors contributed equally to these work

| **Table of Contents** | |
| --- | --- |
| Title of paper, author’s names, address | S-1 |
| Synthesis of valienamine **(6)** (Scheme S-1) | S-4 |
| Experimental Methods | S-5 |
| Table S1: ^13^C-NMR comparison of the reported values (enzymatic synthesis and chemical synthesis) and the obtained data for valienamine **(6)** | S-11 |
| ^1^H and ^13^C NMR of (3*R*,4*R*,5*S*)-4,5,7-tris(benzyloxy)-6-oxohept-1-en-3-yl acetate (15) | S-12 |
| ^1^H and ^13^C NMR of (4*S*,5*R*,6*R*)-4,5,6-tris(benzyloxy)-3-((benzyloxy)methyl)octa-1,7-dien-3-ol (**16A**) | S-13 |
| ^1^H and ^13^C NMR of (1*S*,4*S*,6*S*)-4,5,6-tris(benzyloxy)-1-((benzyloxy)methyl)cyclohex-2-en-1-ol (**17A**) | S-14 |
| ^1^H and ^13^C NMR of (1*S*,4*S*,6*S*)-4,5,6-tris(benzyloxy)-1-((benzyloxy)methyl)cyclohex-2-en-1-yl carbamate (**18**) | S-15 |
| ^1^H and ^13^C NMR of Benzyl((1*S*,4*R*,6*S*)-4,5,6-tris(benzyloxy)-3-((benzyloxy)methyl)cyclohex-2-en-1-yl)carbamate (**19**) | S-16 |
| ^1^H and ^13^C NMR of (1*S*,3*R*,6*S*)-6-amino-4-(hydroxymethyl)cyclohex-4-ene-1,2,3-triol (**6**) (Valienamine) | S-17 |
| ^1^H and ^13^C NMR of 3,4,7-Tri-*O*-benzyl-5-*O*-(2’,3’,4’,6’-tetra-*O*-benzyl-*α*-D-glucopyranosyl)-D-gluchept-1-enone (**26**) | S-18 |
| ^1^H and ^13^C NMR of 3,4,9-Tri-*O*-benzyl-5-*O*-(2’,3’,4’,6’-tetra-*O*-benzyl-*α*-D-glucopyranosyl)-D-gluco-octa-1,7-dienitol (**27A**) | S-19 |
| ^1^H and ^13^C NMR of 3,4,9-Tri-*O*-benzyl-5-*O*-(2’,3’,4’,6’-tetra-*O*-benzyl-*α*-D-glucopyranosyl)-L-ido-octa-1,7-dienitol (**27B**) | S-20 |
| ^1^H and ^13^C NMR of (1D)-(1,3,4/2)-1,2-Di-*O*-benzyl-4-*C*-[(benzyloxy)methyl]-3-*O*-(2’,3’,4’,6’-tetra-*O*-benzyl-*α*-D-glucopyranosyl)cyclohex-5-ene-1,2,3,4-tetrol (**28A**) | S-21 |
| ^1^H and ^13^C NMR of (1D)-(1,3,4/2)-1,2-Di-*O*-benzyl-4-*C*-[(benzyloxy)methyl]-3-*O*-(2’,3’,4’,6’-tetra-*O*-benzyl-*α*-D-glucopyranosyl)cyclohex-5-ene-1,2,3,4-tetrol (**28B**) | S-22 |
| ^1^H and ^13^C NMR of **(**1D)-(1,3,4/2)-1,2-Di-*O*-benzyl-4-*C*-[(benzyloxy)methyl]-4-*O*-carbamoyl-3-*O*-(2’,3’,4’,6’-tetra-*O*-benzyl-*α*-D-glucopyranosyl)cyclohex-5-ene-1,2,3,4-tetrol (**29**) | S-23 |
| ^1^H and ^13^C NMR of (*1*D)-(1,3,4/2)-1,2-Di-*O*-benzyl-6-[(benzyloxycarbonyl) amino]-4-[(benzyloxy)methyl]-3-*O*-(2’,3’,4’,6’-tetra-*O*-benzyl-*α*-D-glucopyranosyl) cyclohex-4-ene-1,2,3-triol (**30**) | S-24 |
| ^1^H and ^13^C NMR of L-chiro-Inisitol-1-amino-1,5,6-trideoxy-4-*O*-(*α*-D-glucopyranosyl)-5-(hydroxymethyl) or 4-*α*-glycoside derivative of validamine (**7**) | S-25 |
| ^1^H and ^13^C NMR of *α*-D-Glucopyranoside,4-amino-5,6-dihydroxy-2-(hydroxymethyl)-2-cyclohexen-1-yl or 4-*α*-glycoside derivative of valienamine (**8**) | S-26 |
| Figure S1: Superimposed active sites of Apo *Sco* GlgE1-V279S (magenta carbon atoms), *Sco* GlgE1-V279S/**7** and *Sco* GlgE1-V279S/**8** structures (colored as in previous figures). | S-27 |
| Table S1: Crystallographic Data Table for *Sco* GlgE1-V279S/**7** (7MEL) *Sco*GlgE1-V279S/**8** (7MGY) | S-28 |
| References | S-29 |

**Synthesis of Valienamine (6)**

Scheme S1. Reagents and conditions: (i) Ac_2_O, pyridine, cat. DMAP, rt (95%); (ii) BF_3_-Et_2_O, 4-Methyl thiophenol, CH_2_Cl_2_, rt; (iii) NaOMe, MeOH, 1 h, rt (90%); (iv) NaH, BnBr, DMF, rt (92%); (v) NBS, Acetone:Water (9:1), rt (88%); (vi) *n*BuLi, Ph_3_PCH_3_Br, dry THF, rt (75%); (vii) Toluene, DMSO, pyridine,TFA, DCC, rt, (81%); (viii) VinylMgBr, THF, –78 ̊C, 2 h (9:1) (82%); (ix) Grubb's 1st gen. 40 ̊C, (82%); (x) (a) CCl_3_CONCO, DCM (b) K_2_CO_3_, MeOH/H_2_O, 0 ̊C–rt, (90%); (xi) (a) Et_3_N, TFAA, DCM, –20 ̊C (b) BnOH, NaH, THF, rt, (60%); (xii) Na, Liq. NH_3_, Dry THF, –78 ̊C, quantitative.

The secondary alcohol in **14** was oxidized using modified Moffatt’s oxidation conditions to afford ketone **15** in overall 90% yield.^1^ Compound **15** was converted to valienamine **(6)** in five steps following reported literature.^2^ This synthetic route involved the stereoselective addition of vinyl magnesium bromide in **15** to afford two separable diastereomers **(16A** and **16B)** (9:1). Intramolecular ring closing metathesis (RCM) of the desired diene **(16A)** gave the carbasaccharide **(17A)**. The tertiary alcohol of **17A** was then converted to carbamate **(18)** and subjected to an Ichikawa rearrangement^3^ to give the protected valienamine analog **(19)** in overall 75% yield. Finally, Birch reduction of the compound **19** gave compound **6** in a quantitative yield (scheme 3).^2^

**Experimental Methods**

**1,2,3,4,6, -penta-*O*-acetyl-D-glucose (10)^4^**

D-(+)- glucose monohydrate (1.55 g, 4.50 mmol) was suspended in dry pyridine (5.70 mL, 69 mmol) and acetic anhydride (4.90 mL, 51.8 mmol) and a catalytic amount of 4-dimethylaminopyridine was added. The solution was stirred at ambient temperature for 16 h. The reaction mixture was diluted with ethyl acetate and washed successively with 1 N HCl (20 mL) and saturated aq. NaHCO_3_ (40 mL). The resulting organic phase was dried (anhydrous Na_2_SO_4_), filtered and the filtrate was concentrated under reduced pressure to afford **10**: yield 95% (2.80 g). All the NMR values match with the reported data.^4^

**4-methylthiophenyl-*β*-D-glucoside (11)^4^**

Peracetylated glucose **10** (2.80 g, 4.30 mmol) was dissolved in dichloromethane, 4-Methylthiophenol (1.10 g, 8.80 mmol) and boron trifluoride diethyl etherate (7.50 mL, 61.0 mmol) were added to the solution at 0 ̊ ºC. The resulting solution was stirred at ambient temperature under N_2_ atmosphere. The reaction was monitored by TLC and appeared to be complete after 4 h. The reaction mixture was diluted with dichloromethane (30 mL) and successively washed with saturated aq. NaHCO_3_ (45 mL), 10% aq. NaCl (45 mL), and water (35 mL). The resulting organic phase was dried (anhydrous Na_2_SO_4_), filtered and the filtrate was concentrated under reduced pressure. The product was dried overnight and deacetylated by dissolving in dry methanol followed by addition of a catalytic amount of sodium metal until the solution reached pH 9. The reaction was monitored for completion using TLC. The reaction was neutralized by adding Amberlite IRA-118H H+ resin until the pH reached 7. Then, the resin was filtered away and the filtrate concentrated under reduced pressure to afford the 4-methylthiophenyl glucoside **11**: yield 90% (1.29 g). All the NMR values match with the reported data.^4^

**4-methylthiophenyl 2,3,4,6-tetrakis-*O*-benzyl-*β*-D-glucopyranoside (12)^4^**

A solution of **11** (1.29 g, 3.90 mmol) in dry *N,N*-dimethylformamide (100 mL) was cooled to 0 ºC. The solution was treated drop-wise with a suspension of sodium hydride (60% dispersion in mineral oil) (1.97 g, 49.4 mmol) in dry *N,N*-dimethylformamide. Benzyl bromide (5.00 mL, 4.14 mmol) was added drop-wise over 15 min. and the solution stirred at room temperature for 16 h. The reaction was poured over ice and extracted with ethyl acetate (50 mL). The combined organic layers were washed with brine. The resulting organic phase was dried (anhydrous Na_2_SO_4_), filtered and the filtrate was concentrated under reduced pressure to obtain a product. The product was purified by silica gel flash column chromatography by eluting with 10:1 hexanes:ethyl acetate. The product fractions were combined, concentrated, and dried in vacuum to afford a yellow oily product; yield: 92% (3.60 g). All the NMR values match with the reported data.^4^

**2,3,4,6-tetrakis-*O*-benzyl-*α/β*-D-glucoside (13)^5^**

*N*-Bromosuccinimide (1.30 g, 7.40 mmol) was added to a solution of **12** (3.60 g, 3.60 mmol) in 9: 1 acetone-water (60 mL) and stirred at room temperature for 45 min. The solvent was evaporated at room temperature until turbid, and a solution of the residue in ethyl acetate (100 mL) was washed with satd. aq. NaHCO_3_, (3 X 50 mL) and water (3 X 50 mL), was dried (anhydrous Na_2_SO_4_), and evaporated. The product was purified by silica gel flash column chromatography by eluting with 6:1 hexanes:ethyl acetate. The product fractions were combined, concentrated, and dried in vacuum to afford **13** as yellow oil: yield 88% (3.50 g). All the NMR values match with the reported data.^5^

**(3*R*,4*R*,5*R*)-3,4,5,7-tetrakis-*O*-benyl-D-glucohept-1-enitol (14)^6^**

2.22 M *n*-Butyl lithium in hexanes (8.20 mL, 17.9 mmol) was added drop-wise to a suspension of methyltriphenylphosphonium bromide (5.20 g, 14.5 mmol) in tetrahydrofuran (50 mL) at –20 ºC. The solution was stirred at –20 ºC for 15 min. and raised to ambient temperature for 1 h. The solution was cooled to –20 ºC and compound **13** (3.50 g, 3.20 mmol) in 50 mL of tetrahydrofuran was added drop-wise. The solution was stirred at –20 ℃ for 15 min. and allowed to warm to ambient temperature and stirred for 6 h. The solution was diluted with acetone (20 mL) and stirred for 30 min. Diethyl ether (50 mL) was added to precipitate triphenylphosphine oxide. The latter was removed by filtration through Celite™ 545 filter aid. The filtrate was washed successively with saturated aq. NaHCO_3_ and brine (50 mL X 3). The solution was dried (anhydrous Na_2_SO_4_), filtered and filtrate concentrated under reduced pressure to obtain the crude product. The product was purified by silica gel flash column chromatography by eluting with 9:1 hexanes:ethyl acetate. The product fractions were combined, concentrated, and dried in vacuum to afford a yellow oil **14**: yield 75% (1.33 g). All the NMR values match with the reported data.^6^

**(3*R*,4*R*,5*S*)-4,5,7-tris(benzyloxy)-6-oxohept-1-en-3-yl acetate (15)^7^**

Compound **14** (1.33 g, 2.40 mmol) was dissolved by gentle warming in anhydrous toluene (20.0 mL) to this dry dimethyl sulfoxide (20.0 mL) was added. To the clear solution were added anhydrous pyridine (20 mL, 2.40 mmol), trifluoroacetic acid (94.0 µL, 1.20 mmol) and *N,N'-*Dicyclohexylcarbodiimide (1.52 g, 3.60 mmol) in that order. The reaction is left at room temperature for 18 h. After the completion of reaction as monitored by TLC, toluene (20 mL) was added, the crystalline dicyclohexylurea was removed by filtration and washed with toluene. The combined filtrates and washings were extracted with water (20 mL X 3) to remove dimethyl sulfoxide. The organic layer was dried over anhydrous Na_2_SO_4_, evaporated under reduced pressure and subjected to flash column chromatography on silica gel to give product as colourless viscous liquid **15**: yield 81% (1.00 g). All the NMR values match with the reported data.^7^

**(4*S*,5*R*,6*R*)-4,5,6-tris(benzyloxy)-3-((benzyloxy)methyl)octa-1,7-dien-3-ol (16A,16B)^2^**

To a cooled (–78 ºC) solution of **15** (1.00 g, 1.95 mmol) in tetrahydrofuran (30 mL) 0.7 M vinylmagnesium bromide (600 µL, 3.90 mmol) in tetrahydrofuran was added dropwise. The reaction mixture was stirred for 1 h. at the same temperature. The reaction mixture was warmed to room temperature, diethyl ether (50 mL) and aq. NH_4_Cl (50 mL) were added to the reaction mixture. The organic layer was separated and washed with brine (50 mL X 2), dried over anhydrous Na_2_SO_4_. The solvent was evaporated under reduced pressure and the isomers were separated by flash column chromatography on silica gel to afford product as colourless viscous liquid **16A:** yield 70% (770 mg) **16B:** yield 7.0% (77 mg). silica gel TLC *R_f_* = 0.75 and 0.68 (30% EtOAc:Hexane) respectively. Compound **16A**: ^1^H NMR (600 MHz, CDCl_3_) δ 7.38–7.24 (m, 20H), 5.98 (dd, *J* = 17.3, 10.8 Hz, 1H, H-7), 5.90–5.80 (m, 1H, H-2), 5.49 (dd, *J* = 17.3, 1.9 Hz, 1H, H-8a), 5.32 (ddd, *J* = 10.4, 1.8, 0.7 Hz, 1H, H-1a), 5.25–5.17 (m, 2H, H-1b, H-8b), 4.86–4.80 (m, 2H, PhCH), 4.68–4.60 (m, 4H, PhCH), 4.53–4.41 (m, 4H, PhCH), 4.36 (m, 3H, PhCH), 4.08 (dd, *J* = 7.9, 6.2 Hz, 1H, H-3), 3.87 (dt, *J* = 10.5, 3.4 Hz, 2H, H-4, H-5), 3.78 (s, OH), 3.65 (d, *J* = 8.6 Hz, 1H, H-9a), 3.30 (d, *J* = 8.7 Hz, 1H, H-9b). Compound **16B**: ^1^H NMR (600 MHz, CDCl_3_) δ 7.38–7.24 (m, 20H), 6.07 (dd, *J* = 17.4, 10.9 Hz, 1H, H-7), 5.94 (m, 1H, H-2), 5.45 (dd, *J* = 17.4, 2.2 Hz, 1H, H-8a), 5.26 (dd, *J* = 17.1 Hz, 1.0 Hz, 1H, H-1a), 5.25 (dd, *J* = 10.6, 1.0 Hz 1H, H-1b),5.23 (dd, *J* = 10.9, 2.2 Hz 1H, H-8b), 4.86–4.80 (m, 2H, PhCH), 4.68–4.60 (m, 4H, PhCH), 4.53–4.41 (m, 4H, PhCH), 4.36 (m, 3H,PhCH), 4.09 (dd, *J* = 7.5, 4.4 Hz, 1H, H-3), 3.99 (d,*J* = 5.9 Hz, 1H, H-5), 3.74–3.68 (m, 2H, H-4, H-9a), 3.28 (d, *J* = 9.3 Hz, 1H, H-9b), 3.09 (s, OH). All the NMR values match with the reported data.^2^

**(1*S*,4*S*,6*S*)-4,5,6-tris(benzyloxy)-1-((benzyloxy)methyl)cyclohex-2-en-1-ol (17A, 17B)^2^**

Compound **17A:**^1^H NMR (600 MHz, CDCl_3_) δ 7.41–7.18 (m, 20H), 5.94 (dd, *J* = 10.1, 2.0 Hz, 1H, H-6), 5.71 (dd, *J* = 10.2, 2.1 Hz, 1H, H-5), 4.97–4.85 (m, 3H, PhCH), 4.52–4.45 (m, 2H, PhCH), 4.40 (d, *J* = 12.2 Hz, 1H, PhCH), 4.22 (dt, *J* = 8.0, 2.0 Hz, 1H, H-1), 4.05 (dd, *J* = 10.3, 7.9 Hz, 1H, H-2), 3.78 (d, *J* = 10.3 Hz, 1H, H-3), 3.39–3.27 (m, 2H, H-7a, H-7b), 2.83 (s, 1H, OH). Compound **17B:**^1^H NMR (600 MHz, CDCl_3_) δ 7.41–7.18 (m, 20H), 5.74 (m, 1H, H-6), 5.74 (m, 1H, H-5), 4.97–4.85 (m, 3H, PhCH), 4.52–4.45 (m, 2H, PhCH), 4.40 (d, *J* = 12.2 Hz, 1H, PhCH), 4.20 (dt, *J* = 7.2, 2.0 Hz, 1H, H-1), 4.87 (dd, *J* = 10.3, 7.2 Hz, 1H, H-2), 3.76 (d, *J* = 10.3 Hz, 1H, H-3), 3.63–3.83 (m, 2H, H-7a, H-7b), 2.74 (s, 1H, OH). All the NMR values match with the reported data.^2^

**(1*S*,4*S*,6*S*)-4,5,6-tris(benzyloxy)-1-((benzyloxy)methyl)cyclohex-2-en-1-yl carbamate (18)**^2^

To a cooled (0 ºC) solution of **17A** (0.58 g, 1.00 mmol) in dichloromethane (10 mL) was treated dropwise with trichloroacetyl isocyanate (230 µL, 0.20 mmol). The reaction mixture was stirred for 30 min. at the same temperature and evaporated. The residue was dissolved in methanol (20 mL) and water (3 mL), was cooled to 0 ºC, treated with potassium carbonate (0.26 g, 2.00 mmol), and stirred for 2 h. at that temperature and warmed to room temperature and again stirred for another 2 h. After completion of the reaction methanol was evaporated and the aq. soln. was diluted with water (40 mL) and extracted with dichloromethane (50 mL X 3). The organic layer was separated washed with brine (30 mL X 3), dried over anhydrous Na_2_SO_4_. The solvent was evaporated under reduced pressure and purification was performed by flash column chromatography on silica gel to afford product as colourless liquid **18**: yield 90 % (0.53 g) silica gel TLC *R_f_* = 0.25 (30 % EtOAc:Hexanes).  ^1^H NMR (600 MHz, CDCl_3_) δ 7.41–7.07 (m, 20H), δ 6.36 (dd, *J* = 10.3, 2.0 Hz, 1H, H-6), 5.96 (dd, *J* = 10.2, 2.6 Hz, 1H, H-5), 4.91 (d, *J* = 13.6 Hz, 3H, PhCH), 4.65–4.50 (m, 2H, PhCH), 4.41 (d, *J* = 12.0 Hz, 1H, PhCH), 4.23 (d, *J* = 8.1 Hz, 1H, H-7a),4.20 (dt, *J* = 7.2, 2.2 Hz, 1H, H-1), 4.16 (dd, *J* = 10.0, 7.2 Hz, 1H, H-2), 3.92 (d, *J* = 10.5 Hz, 1H, H-3), 3.86 (d, *J* = 8.3 Hz, 1H, H-7b). All the NMR values match with the reported data.^2^

**Benzyl ((1*S*,4*R*,6*S*)-4,5,6-tris(benzyloxy)-3-((benzyloxy)methyl)cyclohex-2-en-1-yl)carbamate (19)^2^**

To a solution of carbamate **18** (530 mg, 0.90 mmol ) and triethylamine (660 µL, 5.40 mmol) in dry tetrahydrofuran (10 mL) cooled to 0 ºC, trifluoroacetic anhydride (248 µL, 1.80 mmol) was added, and the resulting mixture was slowly warmed to room temperature and stirred for 1 h. In a separate flask, sodium hydride suspension in 60% mineral oil (0.12 g, 3.10 mmol) was added to a solution of benzyl alcohol (210 µL, 1.80 mmol) in dry tetrahydrofuran (10 mL) at 0 ºC. After 1 h, the solution of sodium benzyloxide was added to the generated isocyanate and the progress was followed by TLC. After 24 h, volatiles were removed under reduced pressure. The reaction mixture was diluted with water (50 mL) and extracted with dichloromethane (50 mL X 4). The organic layer was separated washed with brine (50 mL X 2), dried over anhydrous Na_2_SO_4_. The solvent was evaporated under reduced pressure and purification was performed by flash column chromatography on silica gel to afford product as colourless liquid **19:** yield 60% (600 mg) silica gel TLC *R_f_* = 0.60 (30 % EtOAc : Hexanes).  ^1^H NMR (600 MHz, CDCl_3_) δ 7.40–7.34 (m, 8H), 7.34–7.30 (m, 12H), 7.30 (s, 3H), 7.29 (d, *J* = 4.9 Hz, 2H), 5.83 (s, 1H, H-5), 5.11 (dd, *J* = 22.5, 6.8 Hz, 3H), 4.77 (d, *J* = 11.4 Hz, 1H, H-6), 4.71 (d, *J* = 11.1 Hz, 2H), 4.67–4.57 (m, 5H), 4.50 (d, *J* = 11.8 Hz, 1H), 4.42 (d, *J* = 11.8 Hz, 1H), 4.26 (d, *J* = 12.1 Hz, 1H, H-7a), 4.08 (d, *J* = 4.7 Hz, 1H, H-3), 3.92 (d, *J* = 12.1 Hz, 1H, H-7b), 3.82 (dd, *J* = 7.6, 4.7 Hz, 1H, H-1), 3.75 (t, *J* = 6.2 Hz, 1H, H-2). All the NMR values match with the reported data.^2^

**(1*S*,2*S*,3*R*,6*S*)-4-Cyclohexene-1,2,3-triol, 6-amino-4-(hydroxymethyl) (Valienamine) (6)**

Ammonia was condensed into a solution of **19** (100 mg, 0.57 mmol) in tetrahydrofuran (5 mL) using a dry ice cooled cold finger apparatus. The solution was treated with sodium in small pieces, until a blue colour in the solution persisted. After stirring for 10 min. at –78 ºC, the mixture was treated with methanol (5 ml), stirred at room temperature and evaporated. The residue was extracted with methanol, filtered and evaporated. The residue (10 mg) was adsorbed on 3 mL of neutral *Dowex 50-WX-8* (washed with water). After washing with water (10 mL), elution with 2% aq. ammonia gave **6** as a white solid (15.0 mg). ^1^H NMR (600 MHz, D_2_O): δ 5.73 (s, 1H), 4.18 (s, 2H), 4.11 (d, *J* = 7.9 Hz, 1H), 4.03 (s, 1H), 3.94 (d, *J* = 10.1 Hz, 1H), 3.68 (d, *J* = 8.9 Hz, 1H).^13^C NMR (600 MHz, D_2_O): δ 145.80, 115.35, 71.56, 70.75, 66.55, 60.86, 49.23. All the NMR values match with the reported data.^2^

**Table S1**: ^13^C-NMR comparison of the reported values (enzymatic synthesis and chemical synthesis) and the obtained data for valienamine **(6)**

**

| C-position | Reported data-isolated^8^ | Data obtained | Reported data-chemical synthesis^9^ |
| --- | --- | --- | --- |
| C-1 | 69.4 | 66.6 | 66.4 |
| C-2 | 74.4 | 71.6 | 71.5 |
| C-3 | 73.6 | 70.8 | 70.7 |
| C-4 | 148.7 | 145.8 | 145.5 |
| C-5 | 118.4 | 115.4 | 115.3 |
| C-6 | 52.1 | 49.2 | 49.2 |
| C-7 | 63.8 | 60.9 | 60.9 |

*ppm from sodium 2,2,dimethyl-2-silapentane sulfonate (DSS) in D_2_O at pD<1*

**^1^H NMR of (3*R*,4*R*,5*S*)-4,5,7-tris(benzyloxy)-6-oxohept-1-en-3-yl acetate (15)**

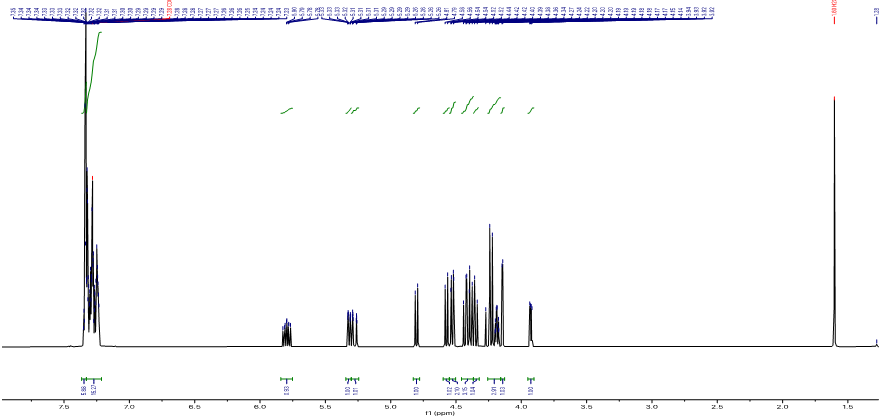


**^13^C NMR of (3*R*,4*R*,5*S*)-4,5,7-tris(benzyloxy)-6-oxohept-1-en-3-yl acetate (15)**


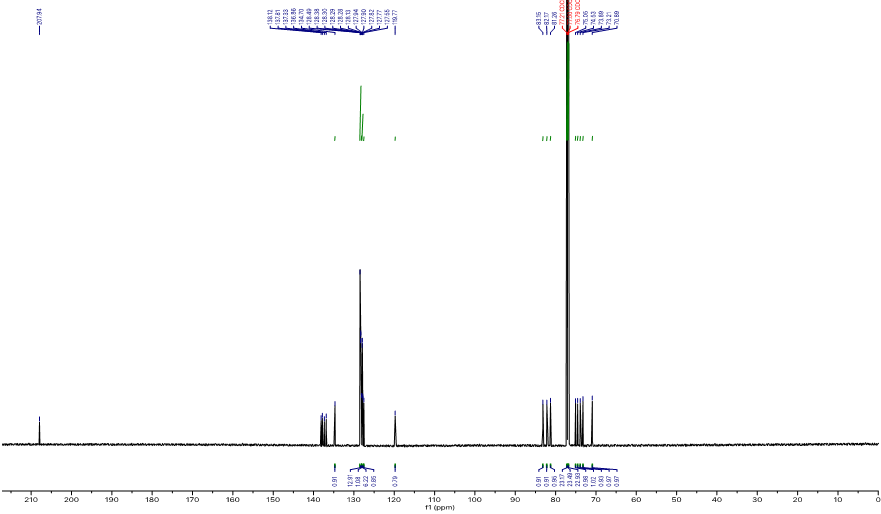


**^1^H NMR of (4*S*,5*R*,6*R*)-4,5,6-tris(benzyloxy)-3-((benzyloxy)methyl)octa-1,7-dien-3-ol (16A)**

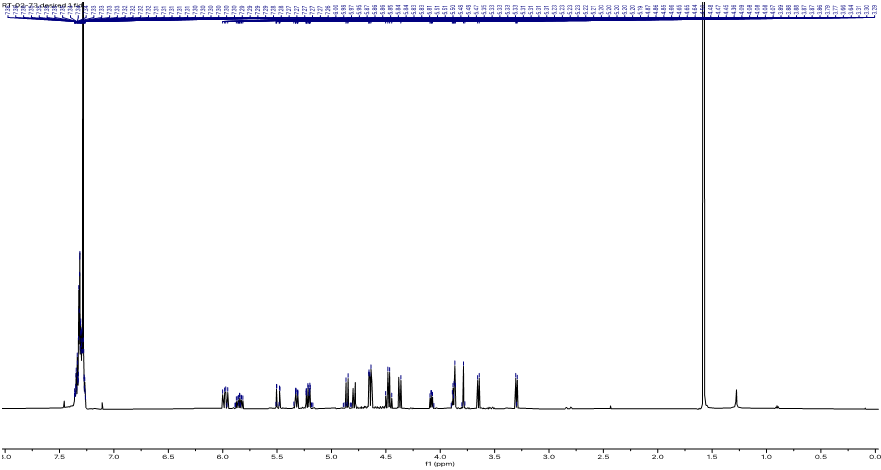


**^13^C NMR of (4*S*,5*R*,6*R*)-4,5,6-tris(benzyloxy)-3-((benzyloxy)methyl)octa-1,7-dien-3-ol (16A)**


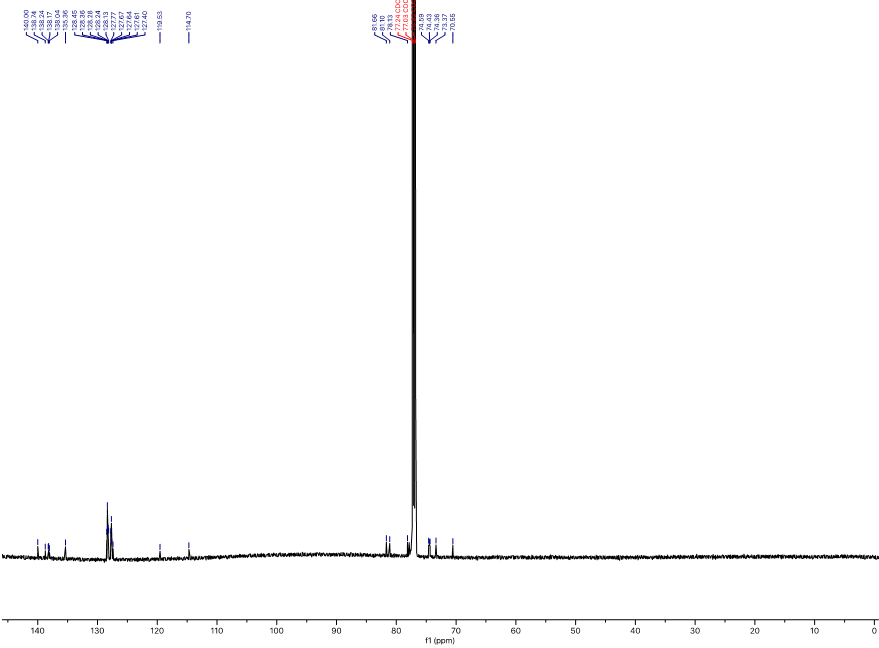


**^1^H NMR of (1*S*,4*S*,6*S*)-4,5,6-tris(benzyloxy)-1-((benzyloxy)methyl)cyclohex-2-en-1-ol (17A)**

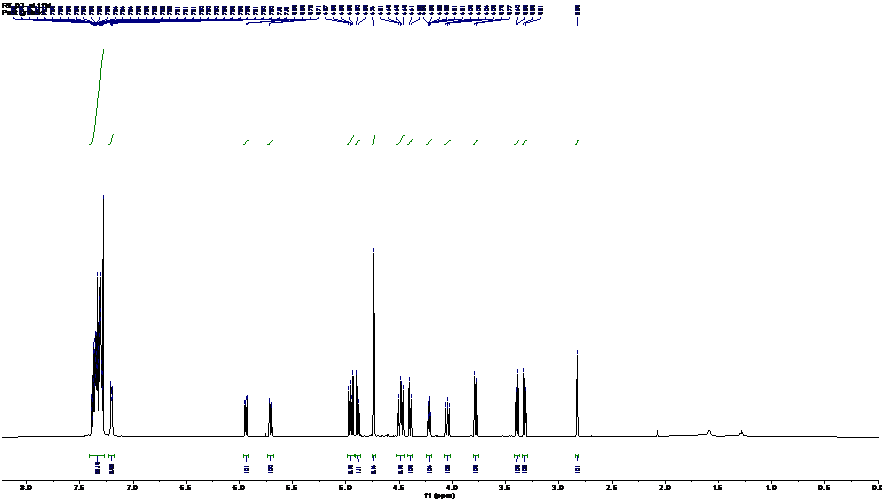


**^13^C NMR of (1*S*,4*S*,6*S*)-4,5,6-tris(benzyloxy)-1-((benzyloxy)methyl)cyclohex-2-en-1-ol(17A)**


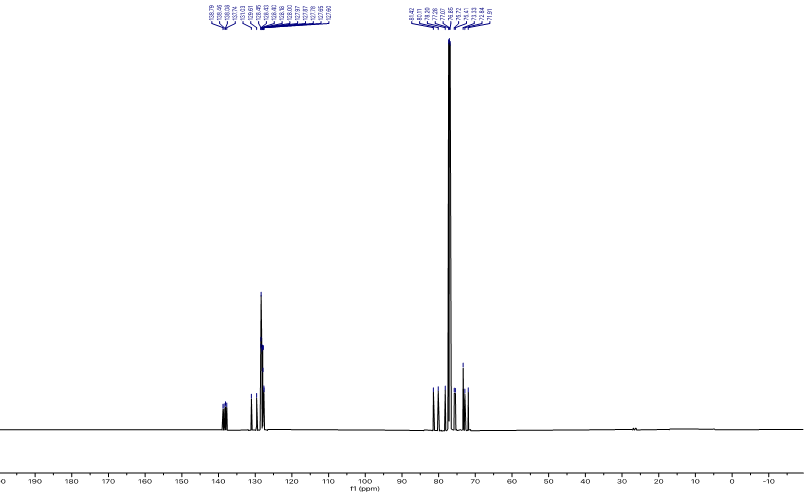


**^1^H NMR of (1*S*,4*S*,6*S*)-4,5,6-tris(benzyloxy)-1-((benzyloxy)methyl)cyclohex-2-en-1-yl carbamate (18)**

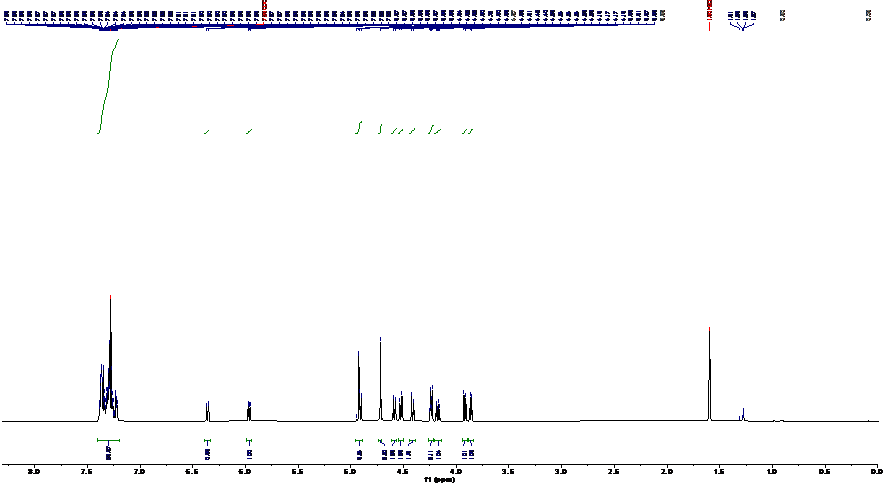


**^13^C NMR of (1*S*,4*S*,6*S*)-4,5,6-tris(benzyloxy)-1-((benzyloxy)methyl)cyclohex-2-en-1-yl carbamate (18)**


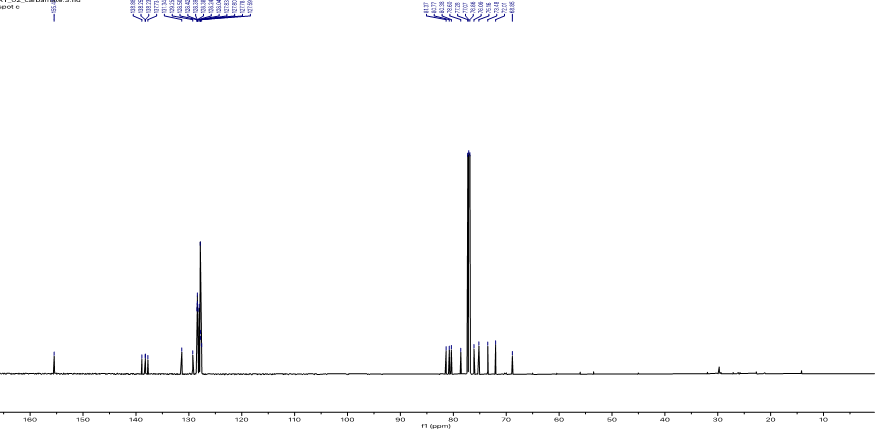


**^1^H NMR of Benzyl((1*S*,4*R*,6*S*)-4,5,6-tris(benzyloxy)-3-((benzyloxy)methyl)cyclohex-2-en-1-yl)carbamate (19)**

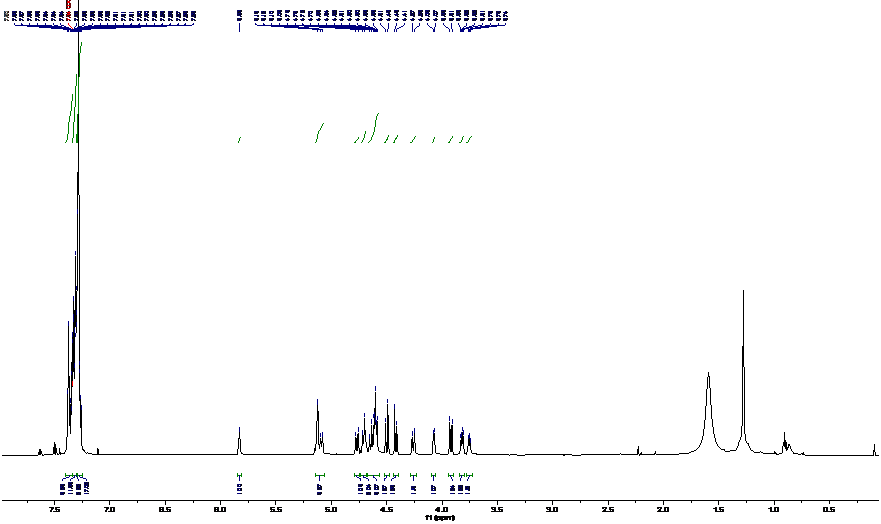


**^13^C NMR of Benzyl((1*S*,4*R*,6*S*)-4,5,6-tris(benzyloxy)-3-((benzyloxy)methyl)cyclohex-2-en-1-yl)carbamate (19)**


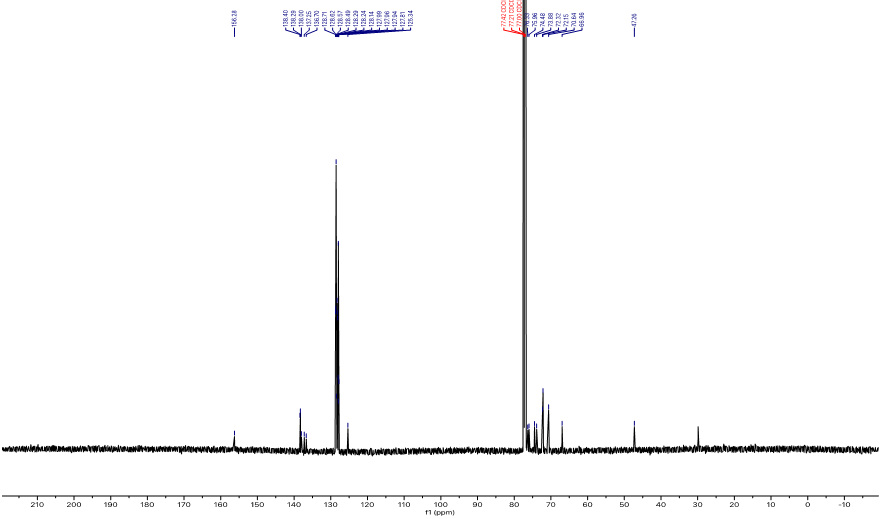


**^1^H NMR of (1*S*,3*R*,6*S*)-6-amino-4-(hydroxymethyl)cyclohex-4-ene-1,2,3-triol (6) (Valienamine)**

**^^**


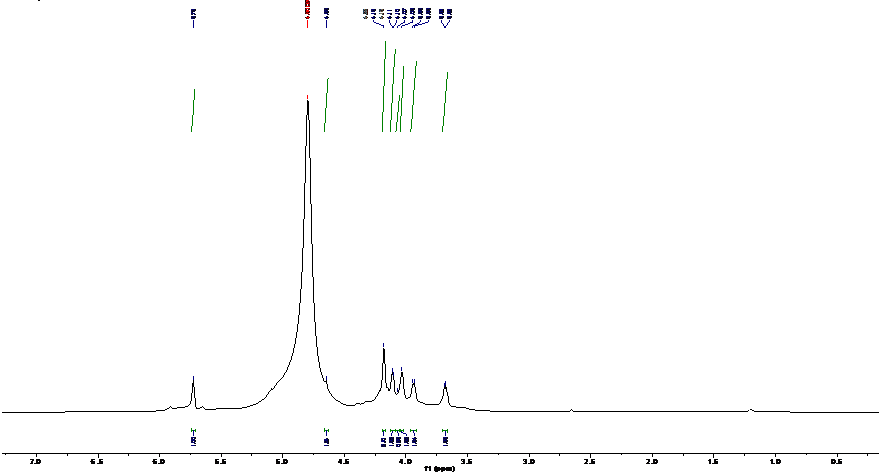


**^13^C NMR of (1*S*,3*R*,6*S*)-6-amino-4-(hydroxymethyl)cyclohex-4-ene-1,2,3-triol (6) (Valienamine)**


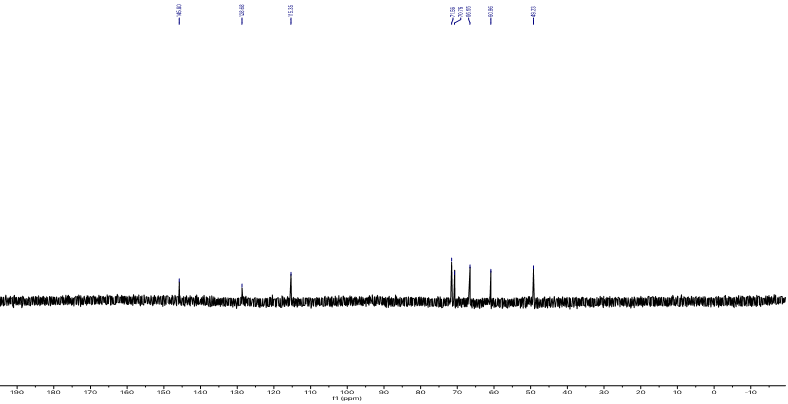


**^1^H NMR of 3,4,7-Tri-*O*-benzyl-5-O-(2’,3’,4’,6’-tetra-*O*-benzyl-*α*-D-glucopyranosyl)-D-gluchept-1-enone (26)**


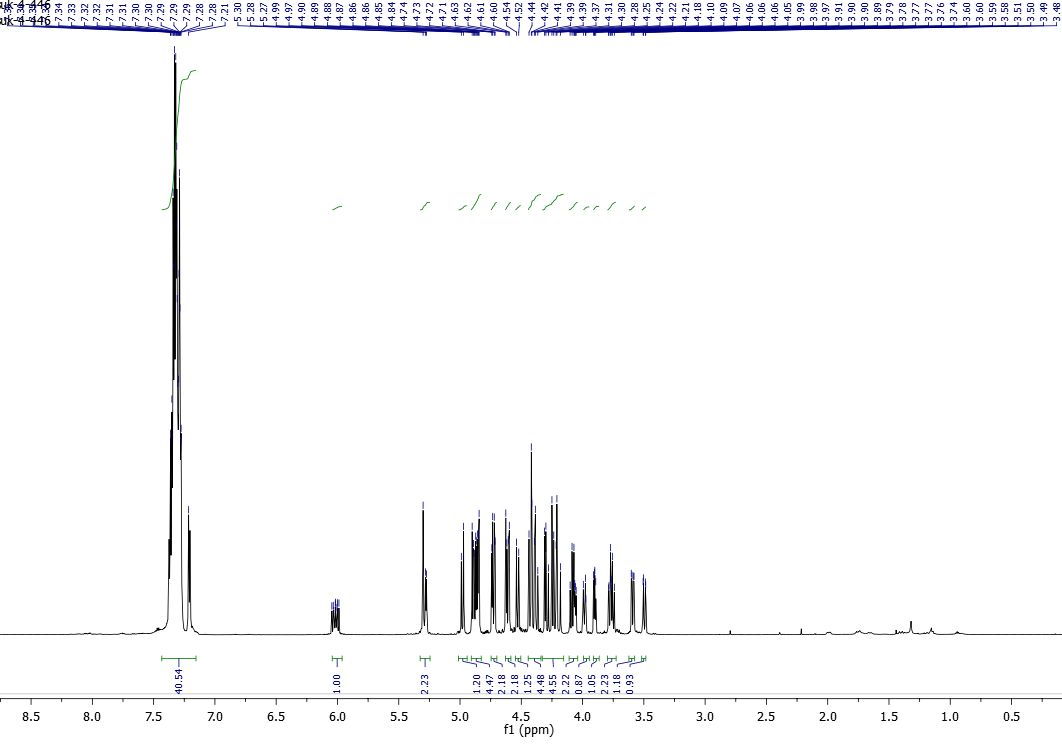


**^13^C NMR of 3,4,7-Tri-*O*-benzyl-5-*O*-(2’,3’,4’,6’-tetra-*O*-benzyl-*α*-D-glucopyranosyl)-D-gluchept-1-enone (26)**


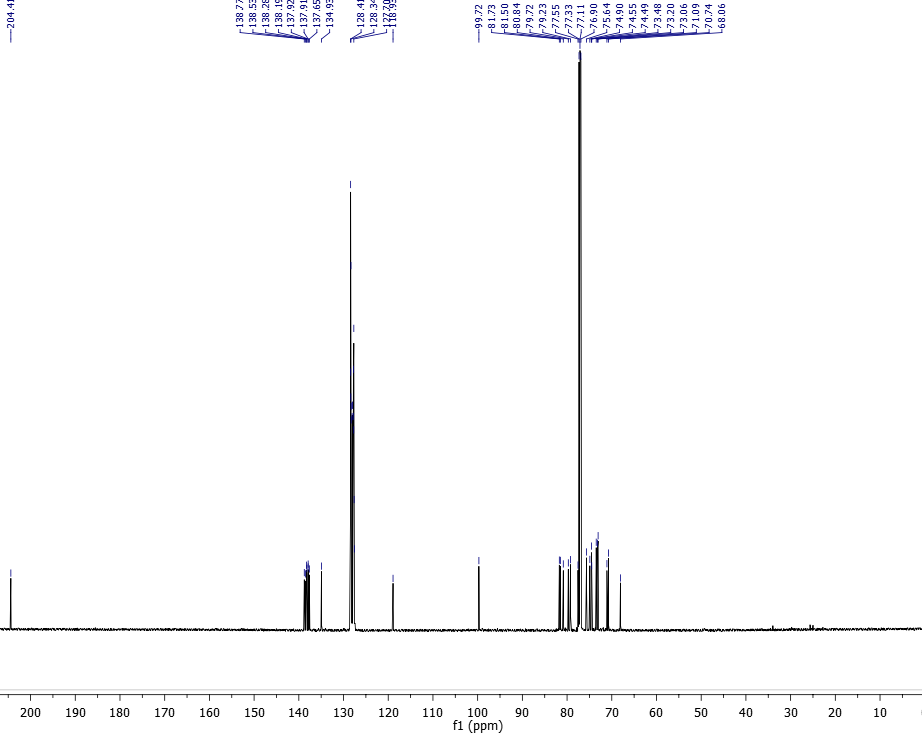


**^1^H NMR of 3,4,9-Tri-*O*-benzyl-5-*O*-(2’,3’,4’,6’-tetra-*O*-benzyl-*α*-D-glucopyranosyl)-D-gluco-octa-1,7-dienitol (27A)**

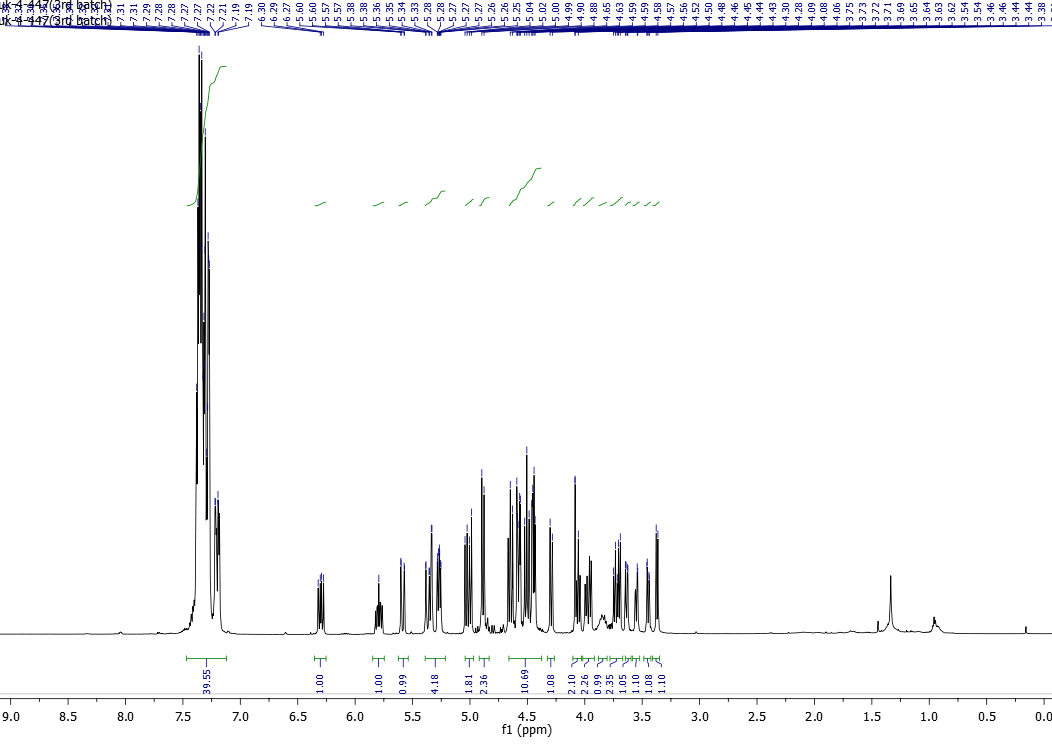


**^13^C NMR of 3,4,9-Tri-*O*-benzyl-5-*O*-(2’,3’,4’,6’-tetra-*O*-benzyl-*α*-D-glucopyranosyl)-D-gluco-octa-1,7-dienitol (27A)**


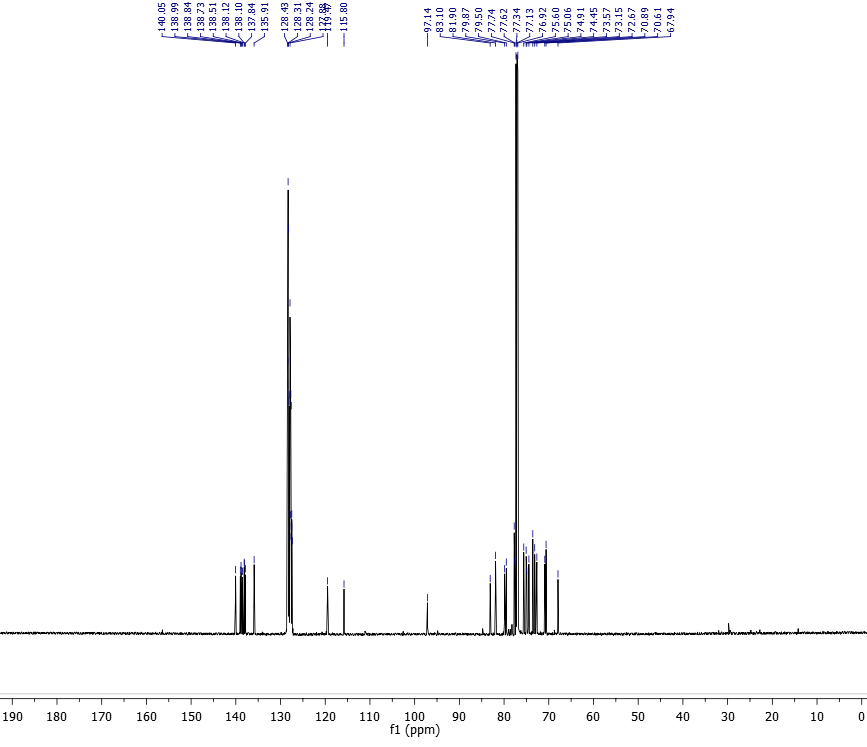


**^1^H NMR of 3,4,9-Tri-*O*-benzyl-5-*O*-(2’,3’,4’,6’-tetra-*O*-benzyl-*α*-D-glucopyranosyl)-L-ido-octa-1,7-dienitol (27B)**


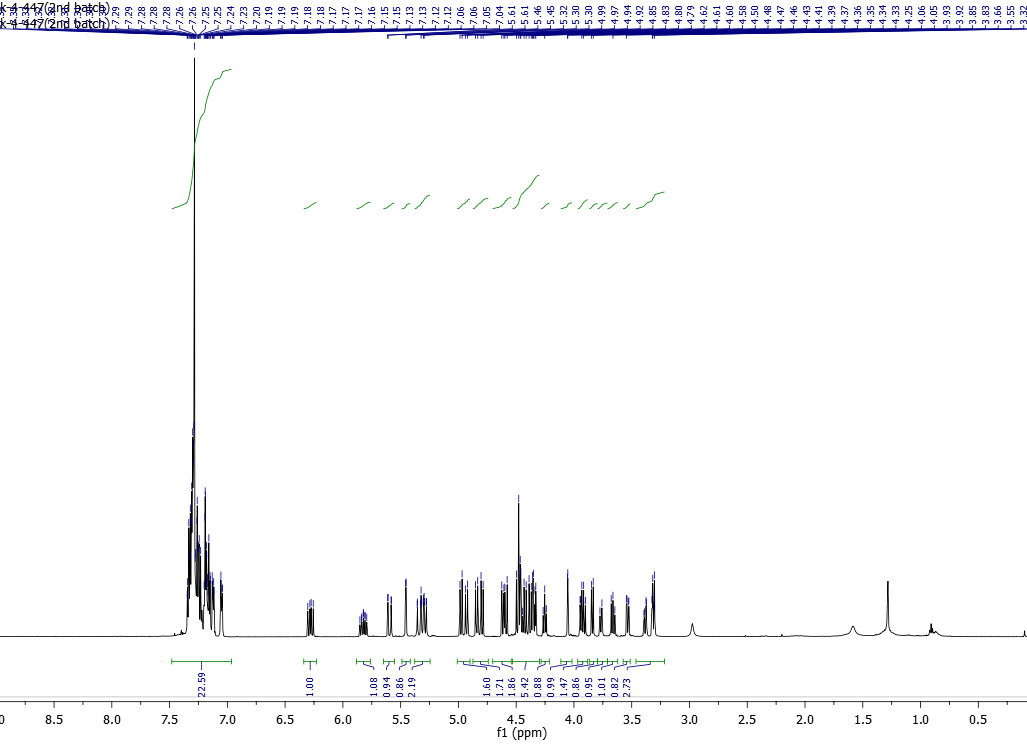


**^13^C NMR of 3,4,9-Tri-*O*-benzyl-5-*O*-(2’,3’,4’,6’-tetra-*O*-benzyl-α-D-glucopyranosyl)-L-ido-octa-1,7-dienitol (27B)**


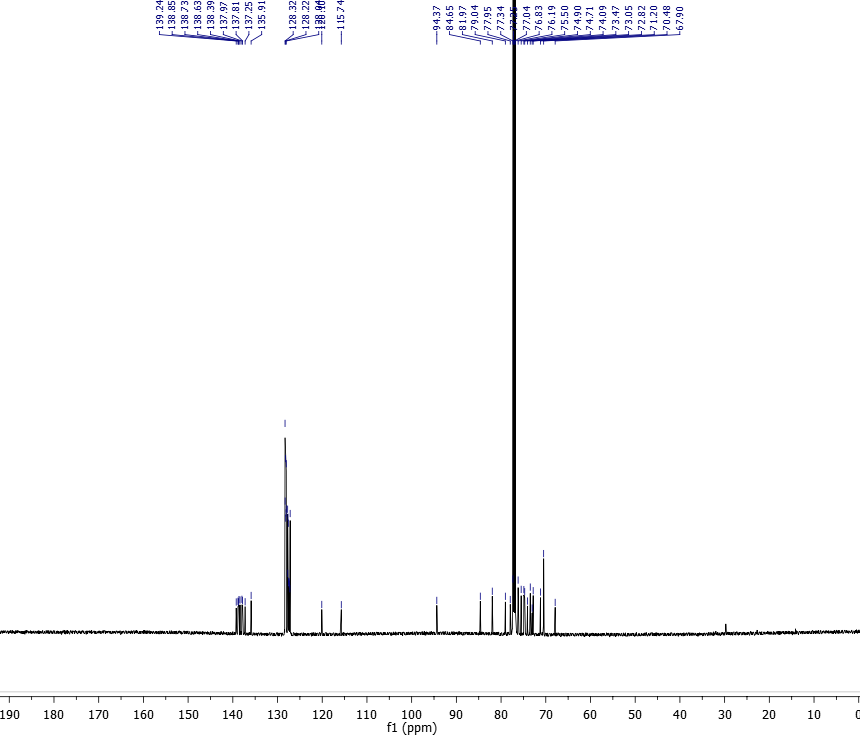


**^1^H NMR of (1D)-(1,3,4/2)-1,2-Di-*O*-benzyl-4-*C*-[(benzyloxy)methyl]-3-*O*-(2’,3’,4’,6’-tetra-*O*-benzyl-*α*-D-glucopyranosyl)cyclohex-5-ene-1,2,3,4-tetrol (28A)**

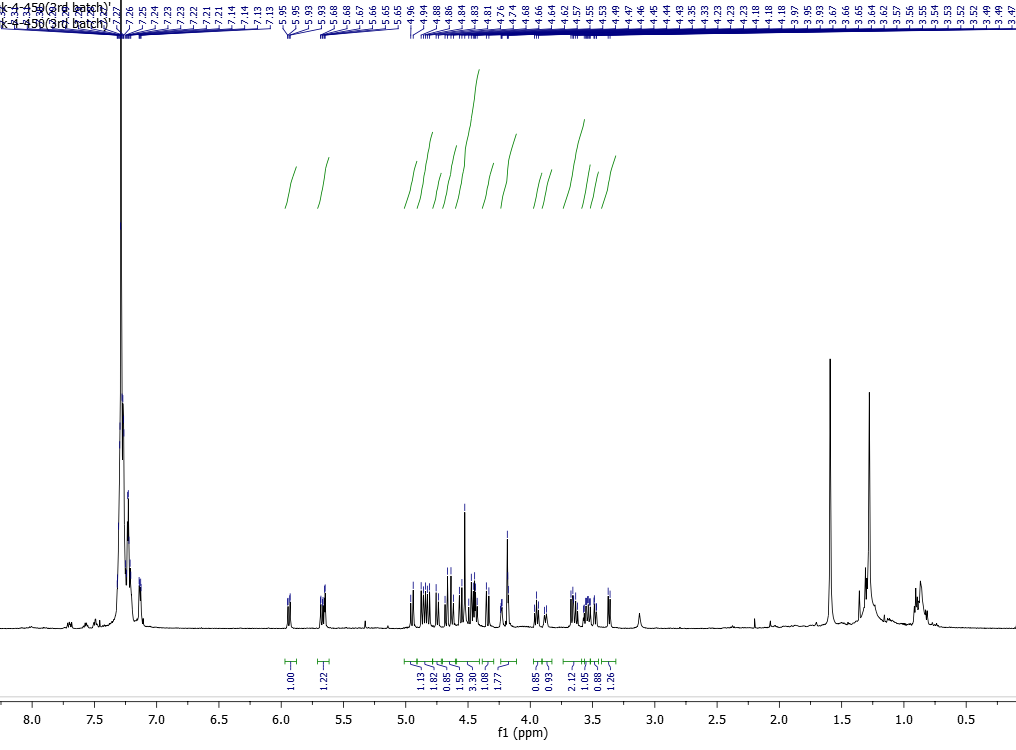


**^13^C NMR of (1D)-(1,3,4/2)-1,2-Di-*O*-benzyl-4-*C*-[(benzyloxy)methyl]-3-*O*-(2’,3’,4’,6’-tetra-*O*-benzyl-*α*-D-glucopyranosyl)cyclohex-5-ene-1,2,3,4-tetrol (28A)**


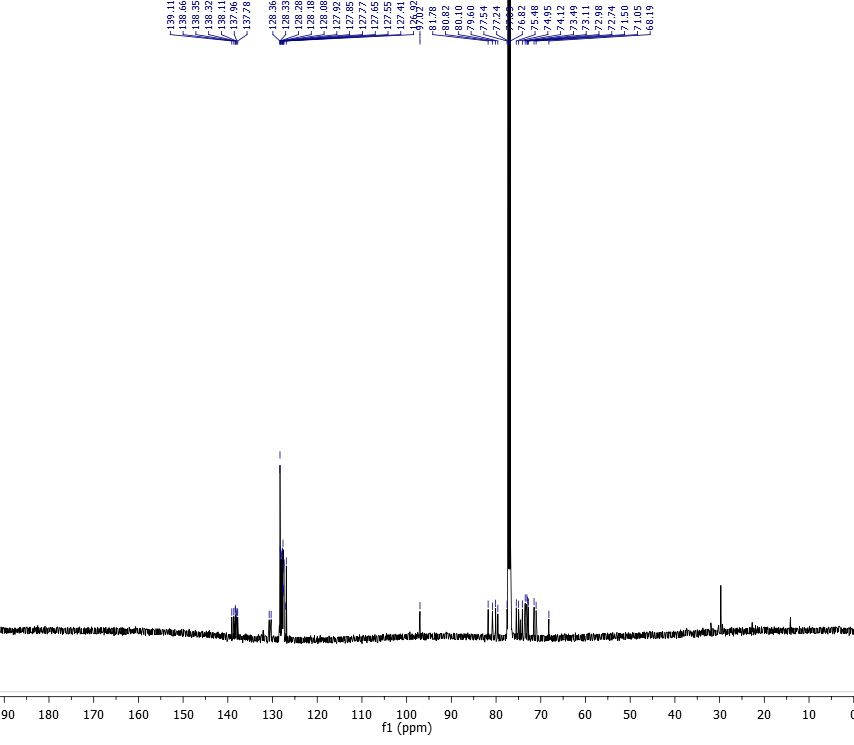


**^1^H NMR of (1D)-(1,3,4/2)-1,2-Di-*O*-benzyl-4-*C*-[(benzyloxy)methyl]-3-*O*-(2’,3’,4’,6’-tetra-*O*-benzyl-*α*-D-glucopyranosyl)cyclohex-5-ene-1,2,3,4-tetrol (28B)**

**^13^C NMR of (1D)-(1,3,4/2)-1,2-Di-*O*-benzyl-4-*C*-[(benzyloxy)methyl]-3-*O*-(2’,3’,4’,6’-tetra-*O*-benzyl-*α*-D-glucopyranosyl)cyclohex-5-ene-1,2,3,4-tetrol (28B)**

**^1^H NMR of (1D)-(1,3,4/2)-1,2-Di-*O*-benzyl-4-*C*-[(benzyloxy)methyl]-4-*O*-carbamoyl-3-*O*-(2’,3’,4’,6’-tetra-*O*-benzyl-*α*-D-glucopyranosyl)cyclohex-5-ene-1,2,3,4-tetrol (29)**

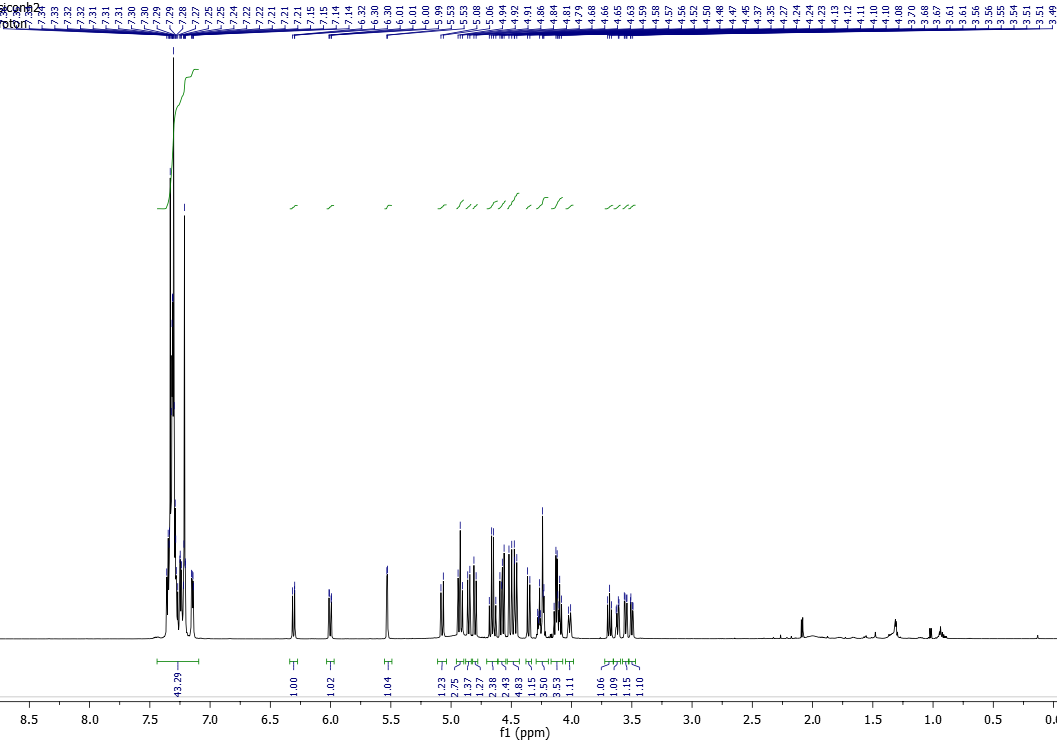


**^13^C NMR of (1D)-(1,3,4/2)-1,2-Di-*O*-benzyl-4-*C*-[(benzyloxy)methyl]-4-*O*-carbamoyl-3-*O*-(2’,3’,4’,6’-tetra-*O*-benzyl-*α*-D-glucopyranosyl)cyclohex-5-ene-1,2,3,4-tetrol (29)**


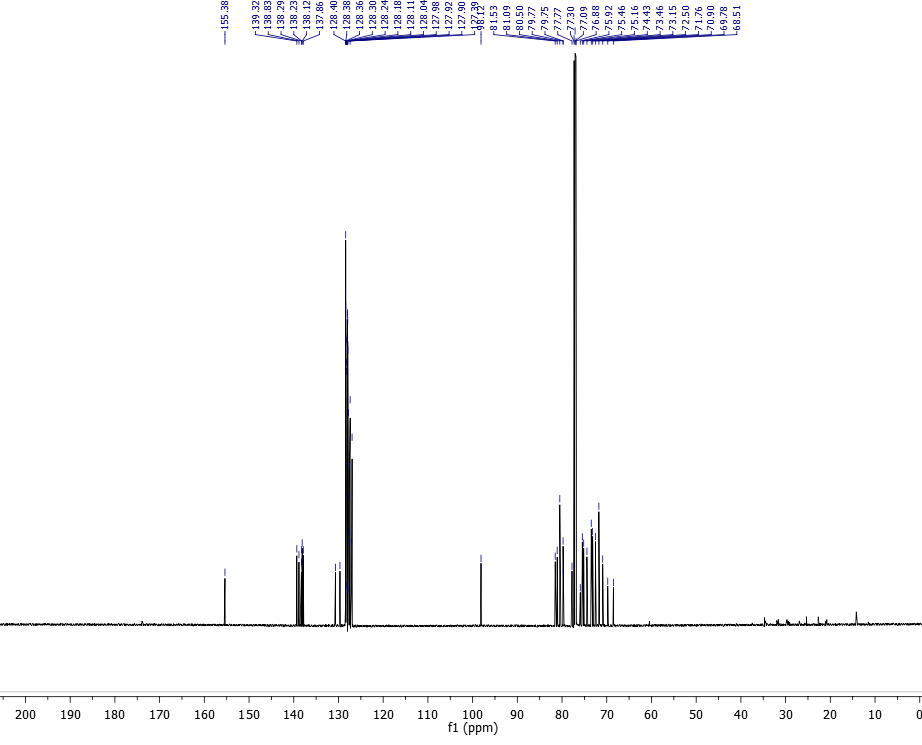


**^1^H NMR of (1D)-(1,3,4/2)-1,2-Di-*O*-benzyl-6-[(benzyloxycarbonyl) amino]-4-[(benzyloxy)methyl]-3-*O*-(2’,3’,4’,6’-tetra-*O*-benzyl-*α*-D-glucopyranosyl) cyclohex-4-ene-1,2,3-triol (30)**

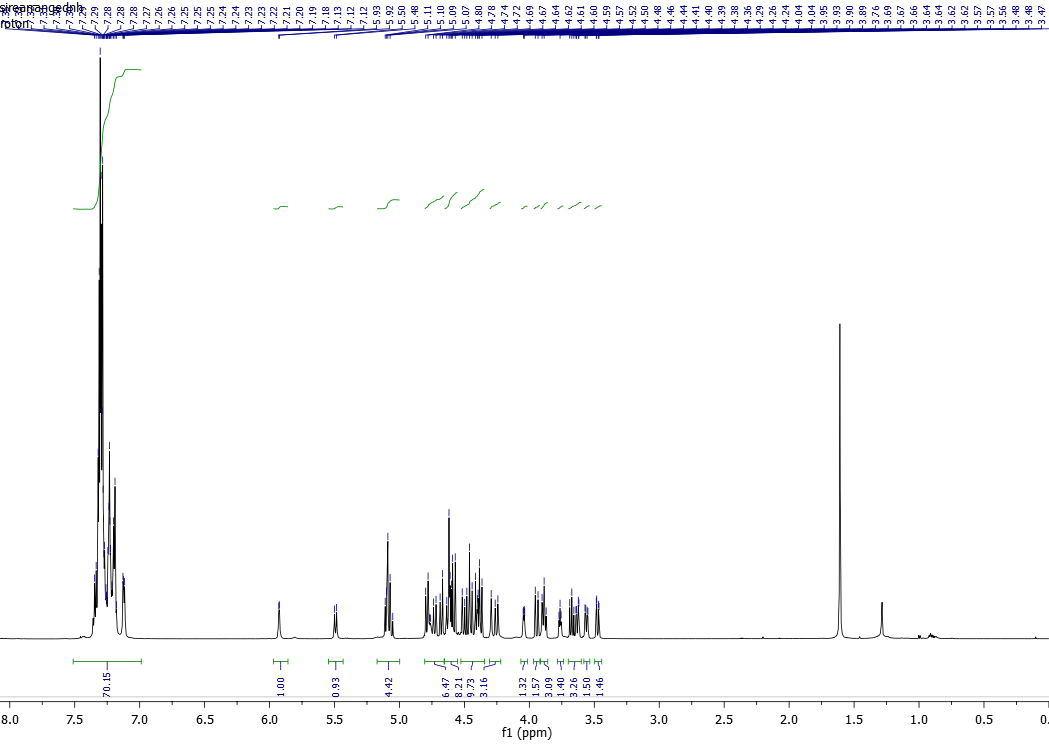


**^13^C NMR of (1D)-(1,3,4/2)-1,2-Di-*O*-benzyl-6-[(benzyloxycarbonyl) amino]-4-[(benzyloxy)methyl]-3-*O*-(2’,3’,4’,6’-tetra-*O*-benzyl-α-D-glucopyranosyl) cyclohex-4-ene-1,2,3-triol (30)**


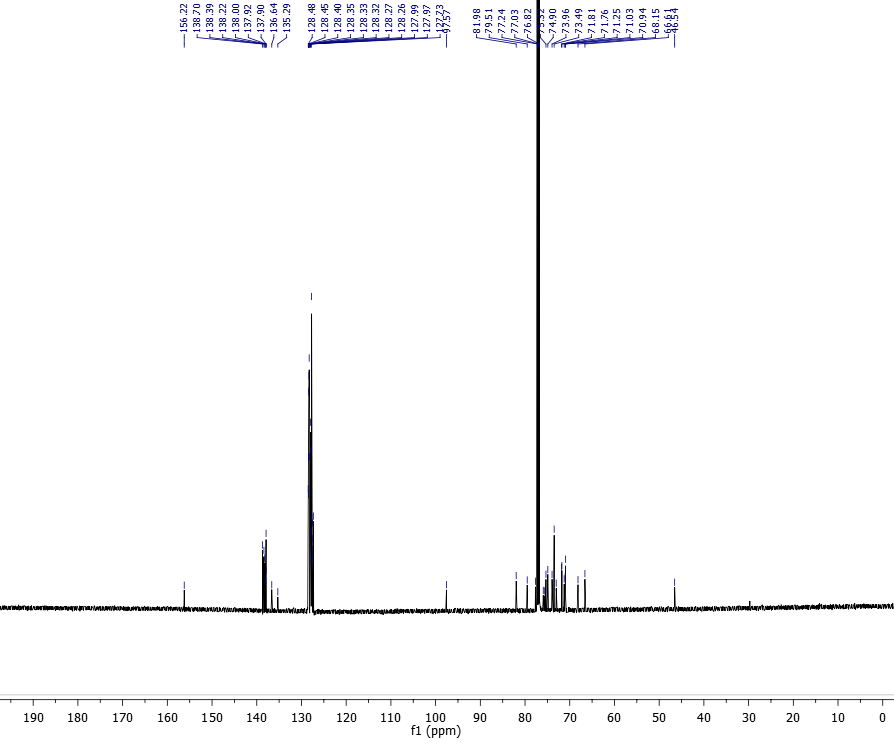


**^1^H NMR of L-chiro-Inisitol-1-amino-1,5,6-trideoxy-4-*O*-(*α*-D-glucopyranosyl)-5-(hydroxymethyl) or 4-*α*-glycoside derivative of validamine (7)**

**
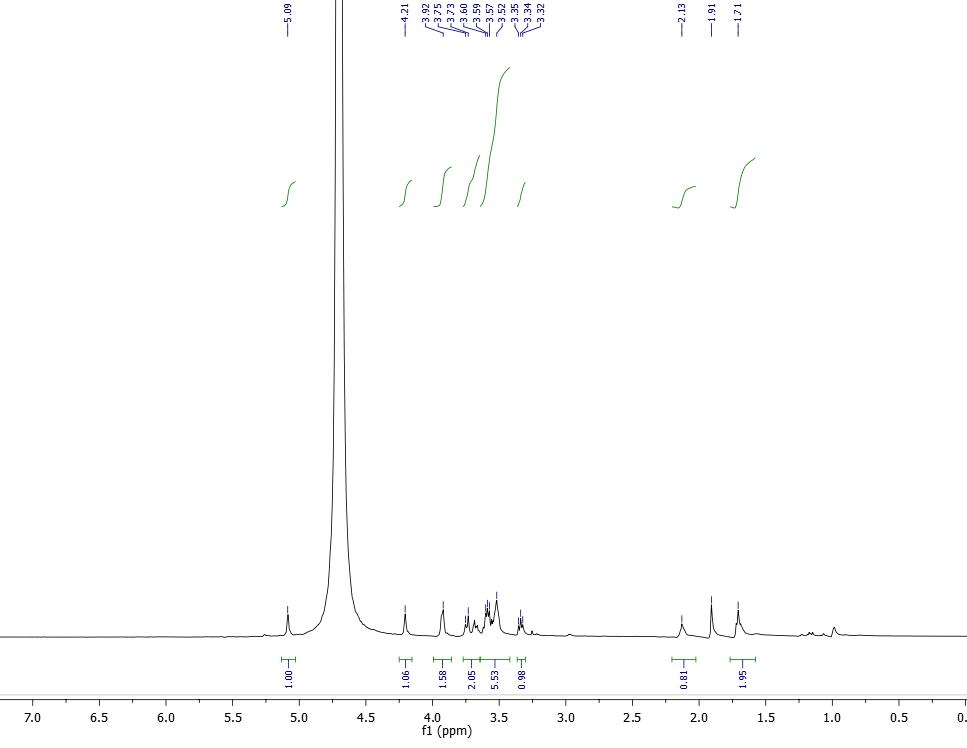
**

**^13^C NMR of L-chiro-Inisitol-1-amino-1,5,6-trideoxy-4-*O*-(*α*-D-glucopyranosyl)-5-(hydroxymethyl) or 4-*α*-glycoside derivative of validamine (7)**


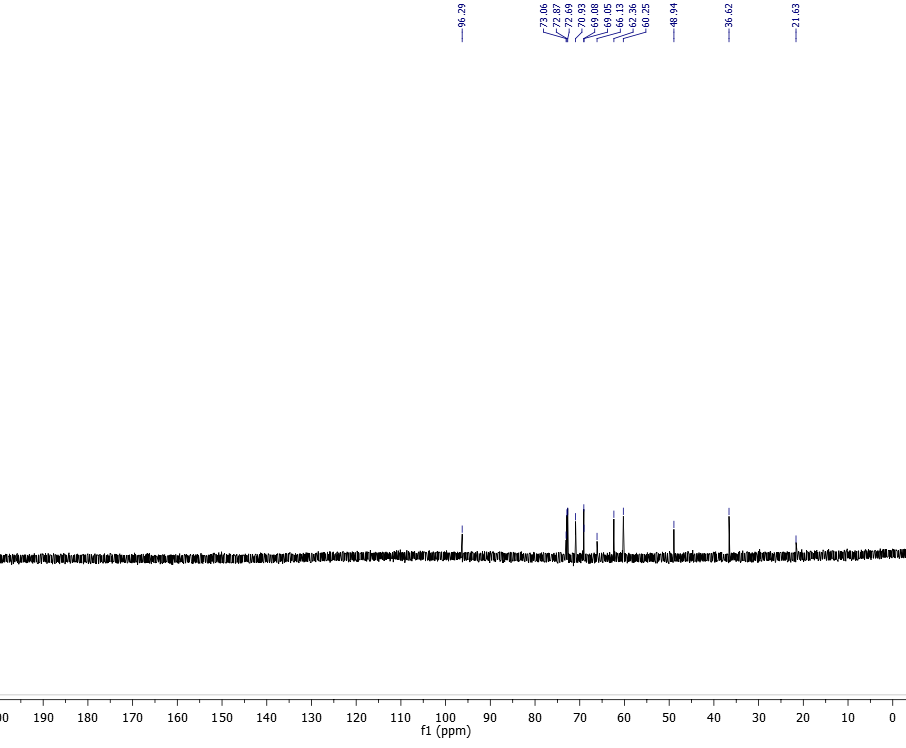


**^1^H NMR of *α*-D-Glucopyranoside,4-amino-5,6-dihydroxy-2-(hydroxymethyl)-2-cyclohexen-1-yl or 4-*α*-glycoside derivative of valienamine (8)**

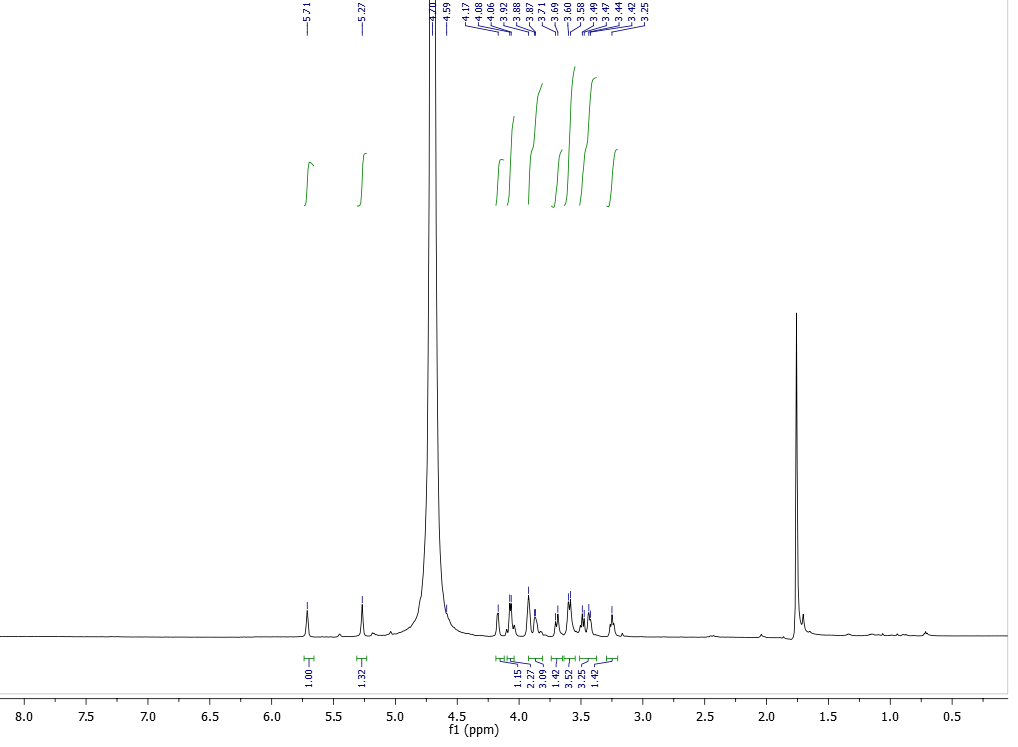


**^13^C NMR of *α*-D-Glucopyranoside,4-amino-5,6-dihydroxy-2-(hydroxymethyl)-2-cyclohexen-1-yl or 4-*α*-glycoside derivative of valienamine (8)**


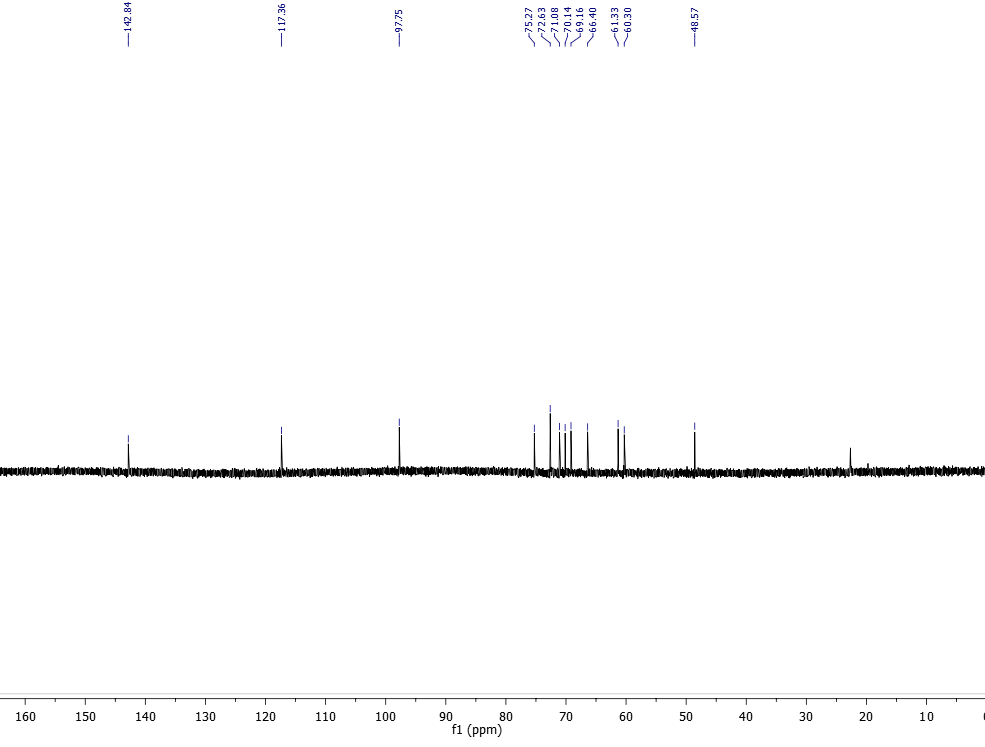


**Figure S1:** Superimposed active sites of Apo *Sco* GlgE1-V279S (magenta carbon atoms), *Sco* GlgE1-V279S/**7** and *Sco* GlgE1-V279S/**8** structures (colored as in previous figures).


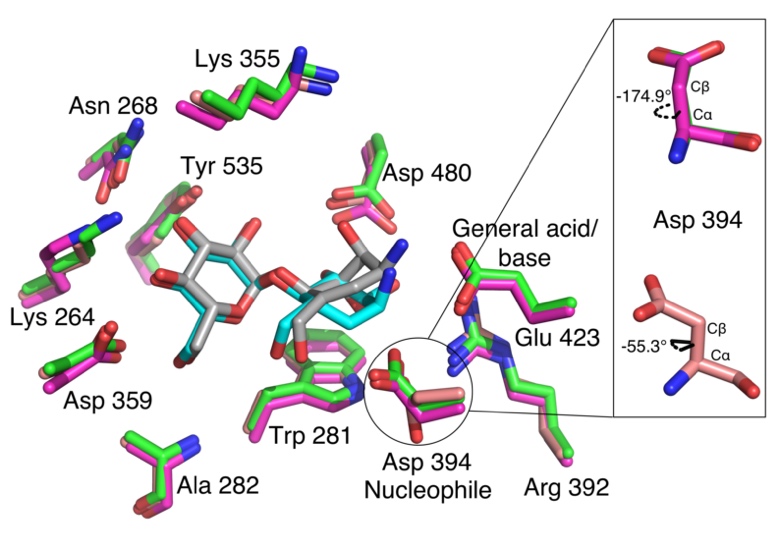


**Table S1**: Crystallographic Data Table for *Sco* GlgE1-V279S/**7** (7MEL) and *Sco*GlgE1-V279S/**8** (7MGY)

|  | *Sco*GlgE1-V279S/**7**  (7MEL) | *Sco*GlgE1-V279S/**8**  (7MGY) |
| --- | --- | --- |
| Data collection | | |
| Wavelength (Å) | 0.9787 | 0.9786 |
| Space group | P 41 21 2 | P 41 21 2 |
| Unit cell dimensions | | |
| a | 113.049 | 112.784 |
| b | 113.049 | 112.784 |
| c | 311.506 | 310.198 |
| α | 90º | 90º |
| β | 90º | 90º |
| ϒ | 90º | 90º |
| Resolution (Å) | 55.62 - 1.75 | 55.48 - 1.83 |
| *R*_merge_ | 0.09187 (1.308) | 0.1802 (6.231) |
| *R*_meas_ | 0.09576 (1.618) | 0.186 (6.499) |
| CC_1/2_ | 0.999 (0.357) | 0.998 (0.436) |
| *I*/σ*I* | 16.20 (0.93) | 9.28 (0.21) |
| Completeness (%) | 96.24 (71.70) | 99.87 (99.25) |
| Refinement | | |
| Total no. of reflections | 2269341 (35997) | 2870781 (213020) |
| No. of unique reflections | 195923 (14983) | 176156 (17390) |
| *R*_work_/*R*_free_ | 0.1762/0.1972 | 0.1856/0.2154 |
| No. of atoms | | |
| Protein | 10267 | 10349 |
| Ligand/ion | 89 | 47 |
| Water | 1602 | 1411 |
| B-factors (Å^2^) | | |
| Protein | 25.83 | 32.88 |
| Ligand/ion | 25.28 | 49.20 |
| Water | 35.32 | 39.73 |
| R.m.s deviations | | |
| Bond length (Å) | 0.008 | 0.008 |
| Bond angels (°) | 0.88 | 0.92 |
| Ramachandran | | |
| Favored (%) | 98.38 | 98.61 |
| Outliers (%) | 0.47 | 0.00 |

**References**

1 Pfitzner, K. & Moffatt, J. A new and selective oxidation of alcohols. *J. Am. Chem. Soc.* **85**, 3027-3028 (1963).

2 Kapferer, P., Sarabia, F. & Vasella, A. Carbasaccharides via ring‐closing alkene metathesis. A Synthesis of (+)‐Valienamine from D‐Glucose. *Helv. Chim. Acta* **82**, 645-656 (1999).

3 Ichikawa, Y. New synthetic method for allylic isocyanates through [3, 3] sigmatropic rearrangement of allylic cyanates. *Synlett* **1991**, 238-240 (1991).

4 Veleti, S. K., Lindenberger, J. J., Thanna, S., Ronning, D. R. & Sucheck, S. J. Synthesis of a poly-hydroxypyrolidine-based inhibitor of *Mycobacterium tuberculosis* GlgE. *J. Org. Chem.* **79**, 9444-9450, (2014).

5 Feng, S. & Li, C. Stereospecific, high-yielding, and green synthesis of β-glycosyl esters. *J. Agric. Food Chem.* **63**, 5732-5739 (2015).

6 Chiara, J. L., Bobo, S. & Sesmilo, E. Stereoselective synthesis of branched cyclopentitols by titanium (III)-promoted reductive cyclization of 4-oxiranylaldehydes and 4-oxiranyl ketones derived from hexoses. *Synthesis* **2008**, 3160-3166 (2008).

7 Liu, P. S. Total synthesis of 2, 6-dideoxy-2, 6-imino-7-O-(β-D-glucopyranosyl)-D-glycero-L-gulo-heptitol hydrochloride. A potent inhibitor of α-glucosidases. *J. Org. Chem.* **52**, 4717-4721 (1987).

8 Furumoto, T., Yoshioka, T., Kamata, K., Kameda, Y. & Matsui, K. Enzymic synthesis of valienamine glucosides and their antibiotic activity. *J. Antibiot.* **44**, 371-373 (1991).

9 Fukase, H. & Horii, S. Synthesis of valiolamine and its *N*-substituted derivatives AO-128, validoxylamine G, and validamycin G via branched-chain inosose derivatives. *J. Org. Chem.* **57**, 3651-3658 (1992).
